# ATR‐dependent ubiquitin‐specific protease 20 phosphorylation confers oxaliplatin and ferroptosis resistance

**DOI:** 10.1002/mco2.463

**Published:** 2023-12-20

**Authors:** Jianing Tang, Guo Long, Desheng Xiao, Shuang Liu, Liang Xiao, Ledu Zhou, Yongguang Tao

**Affiliations:** ^1^ Department of Liver Surgery Xiangya Hospital Central South University Changsha Hunan China; ^2^ National Clinical Research Center for Geriatric Disorders Xiangya Hospital Central South University Changsha Hunan China; ^3^ Department of Pathology Xiangya Hospital Central South University Changsha Hunan China; ^4^ Department of Oncology Institute of Medical Sciences National Clinical Research Center for Geriatric Disorders Xiangya Hospital Central South University Changsha Hunan China; ^5^ Department of Pathology Key Laboratory of Carcinogenesis and Cancer Invasion (Ministry of Education) Xiangya Hospital Central South University Hunan China; ^6^ Cancer Research Institute and School of Basic Medicine NHC Key Laboratory of Carcinogenesis (Central South University) Central South University Changsha Hunan China; ^7^ Department of Thoracic Surgery Hunan Key Laboratory of Early Diagnosis and Precision Therapy in Lung Cancer and Hunan Key Laboratory of Tumor Models and Individualized Medicine Second Xiangya Hospital Central South University Changsha Hunan China; ^8^ Hunan Key Laboratory of Cancer Metabolism Hunan Cancer Hospital and Affiliated Cancer Hospital of Xiangya School of Medicine Central South University Changsha Hunan China

**Keywords:** chemoresistance, ferroptosis, hepatocellular carcinoma, solute carrier family 7 member 11, ubiquitin‐specific peptidase 20

## Abstract

Oxaliplatin (OXA) resistance is a major clinic challenge in hepatocellular carcinoma (HCC). Ferroptosis is a kind of iron‐dependent cell death. Triggering ferroptosis is considered to restore sensitivity to chemotherapy. In the present study, we found that USP20 was overexpressed in OXA‐resistant HCC cells. High expression of USP20 in HCC was associated with poor prognosis. USP20 contributes OXA resistance and suppress ferroptosis in HCC. Pharmacological inhibition or knockdown of USP20 triggered ferroptosis and increased the sensitivity of HCC cells to OXA both in vitro and in vivo. Coimmunoprecipitation results revealed that the UCH domain of USP20 interacted with the N terminal of SLC7A11. USP20 stabilized SLC7A11 via removing K48‐linked polyubiquitination of SLC7A11 protein at K30 and K37. Most importantly, DNA damage‐induced ATR activation was required for Ser132 and Ser368 phosphorylation of USP20. USP20 phosphorylation at Ser132 and Ser368 enhanced its stability and thus conferred OXA and ferroptosis resistance of HCC cells. Our study reveals a previously undiscovered association between OXA and ferroptosis and provides new insight into mechanisms regarding how DNA damage therapies always lead to therapeutic resistance. Therefore, targeting USP20 may mitigate the development of drug resistance and promote ferroptosis of HCC in patients receiving chemotherapy.

## Graphical abstract

USP20 is a key factor in the interaction between oxaliplatin (OXA) resistance and ferroptosis in hepatocellular carcinoma. OXA induced DNA damage activates ATR, which phosphorylates USP20 at Ser132 and Ser368. USP20 phosphorylation confers OXA and ferroptosis resistance of HCC cells. Our study reveals a previously unrecognized link between OXA and ferroptosis and provides new insight into mechanisms regarding how DNA damage therapies always lead to therapeutic resistance.

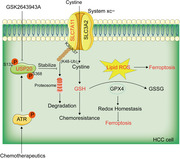

## 1 INTRODUCTION

Hepatocellular carcinoma (HCC) is a commonly diagnosed malignant tumor, which accounts for over 90% of all primary liver cancer cases.^1^ It is an aggressive solid tumor with high malignance.^2^ The 5‐year survival rate of HCC patients is poor and it is still a therapeutic challenge to treat HCC patients.^3^ Thus, it is important to find out novel biomarkers and develop effective therapeutic methods to treat patients with HCC. Oxaliplatin (OXA) is the third generation of platinum anticancer agents, which has been widely applicated in the treatment of different kinds of cancers, including liver, colorectal, pancreatic, and gastric cancer.^4^, ^5^ OXA has been reported to inhibit the growth of liver cancer.^6^ Recent advances of targeted therapy and immunotherapy have improved the therapeutic options of patients, while only a few patients benefit from the targeted therapy and immunotherapy.^7^, ^8^ OXA is still a commonly used chemotherapeutic agent for patients receiving surgical resection.^9^, ^10^, ^11^, ^12^ And in the patients with advanced HCC, it is also used as subsequent maintenance therapy.^13^ In large unresectable HCC, OXA‐based hepatic arterial infusion chemotherapy yielded higher treatment responses and achieved better survival outcomes^12^, ^14^ than transarterial chemoembolization.^15^ The major molecular mechanism of how OXA kills cells is to form platinum‐DNA adducts, thus inducing DNA damage and crosslinks ultimately leading to the death of tumor cells.^16^, ^17^, ^18^, ^19^, ^20^ Although OXA plays a very important role in the treatment of HCC, partial patients do not respond to OXA therapy due to the presence of both intrinsic and acquired resistance, which is a major challenge in the chemotherapy of patients with advanced HCC. And molecular mechanisms involved in OXA resistance of HCC remain poorly defined. Thus, it is of great clinical importance to explore the underlying mechanisms of OXA resistance and provide novel strategies against OXA resistance.

Redox homeostasis is critical for the maintenance of normal cellular activity.^21^ Ferroptosis is a kind of iron‐dependent, programmed cell death, which is induced by the loss of cellular redox homeostasis.^22^, ^23^, ^24^ The initiation of ferroptosis is associated with dysregulated ferroptotic molecular on antioxidant metabolism, iron and lipid metabolism.^25^ The cystine/glutamate antiporter system Xc−/glutathione (GSH)/glutathione peroxidase 4 (GPX4) axis plays a crucial role in regulating ferroptosis. Ferroptosis can be induced by the suppression of system Xc−, GSH, or cysteine depletion.^26^, ^27^, ^28^, ^29^, ^30^ Solute carrier family 7 member 11 (SLC7A11) is the subunit of the Xc− to transport extracellular cystine. Erastin and sorafenib could decrease cysteine transport into cells by inhibiting SLC7A11 and induce ferroptosis.^31^ SLC7A11 is overexpressed in various types of cancers; it is important for the maintenance of intracellular GSH levels which protects cells from oxidative stress. The expression of SLC7A11 is also associated with chemoresistance. Targeting SLC7A11 triggers ferroptosis through lipid peroxidation and Fe^2+^ accumulation and restores sensitivity to chemotherapy.^32^, ^33^, ^34^ While whether SLC7A11 is associated with OXA resistance of HCC remains unclear.

Ubiquitination is an essential posttranslational modification for the maintenance of cellular homeostasis.^35^ Growing studies has revealed that ubiquitination plays crucial roles in the processes such as DNA repair, apoptosis, cell survival, cell‐cycle progression, and antigen presentation.^36^, ^37^ It is of note that protein ubiquitination is dynamic and reversible, which is precisely regulated by the deubiquitylating enzymes (DUBs) and E3 ubiquitin ligases.^38^, ^39^, ^40^ However, the functions of DUBs in the response to OXA therapy in HCC remains unclear.

In the present study, we identified ubiquitin‐specific peptidase 20 (USP20) as a contributor to OXA resistance and suppress ferroptosis in HCC. Pharmacological inhibition or knockdown of USP20 sensitized HCC cells to ferroptosis and reversed OXA resistance both in vitro and in vivo. Targeting USP20 may mitigate the development of drug resistance and promote ferroptosis of HCC in patients receiving chemotherapy.

## 2 RESULTS

### 2.1 Identification of USP20 as an OXA resistance‐related DUB

**FIGURE 1:**
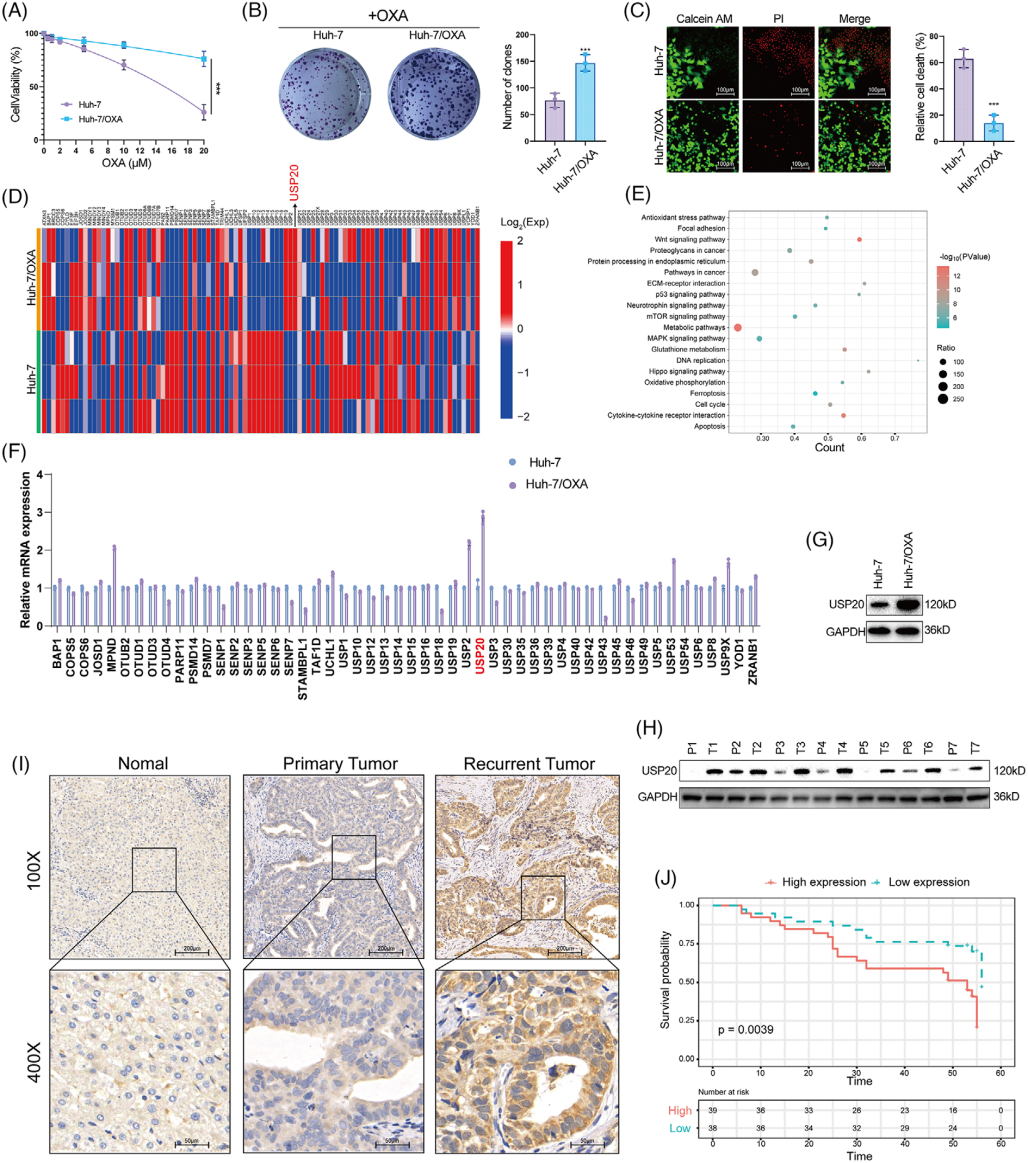
USP20 is upregulated in oxaliplatin (OXA)‐resistant hepatocellular carcinoma (HCC) cells and associated with poor prognosis. (A) The survival percentage of oxaliplatin‐resistant HCC cells (Huh‐7/OXA) and its parental cells treated with increasing concentration of OXA for 48 h. (Each group contained three replicates.) (B) Crystal violet staining of oxaliplatin‐resistant HCC cells (Huh‐7/OXA) and its parental cells treated with OXA. (C) Calcein/PI staining of oxaliplatin‐resistant HCC cells (Huh7/OXA) and its parental cells treated with OXA (10 μM) for 48 h. (D) Heat map shows the representative expression of deubiquitylating enzymes (DUBs). The global gene expression analysis (oxaliplatin‐resistant HCC cells and its parental cells) was based on RNA sequencing platform from BGI (Beijing Genomic Institute). (E) KEGG pathway analysis of the regulated targets in Huh‐7 versus Huh‐7/OXA transcriptome. (F) qRT‐PCR analysis of the significantly expressed DUBs between Huh‐7 and Huh‐7/OXA cells. (G) USP20 was upregulated in Huh‐7/OXA cells. (H) Western blot was used to detect USP20 protein expression level in seven pairs of HCC tissues and paired para‐cancerous tissues. (I). Representative images of IHC of USP20 in normal and tumor tissues derived from HCC patients. All samples were obtained from patients who underwent surgical resection at Xiangya Hospital. (J) High expression of USP20 was associated with poor prognosis. Results shown are representative of three independent experiments. Data are represented as mean ± SD of biological triplicates.*p value < 0.05, **p value < 0.01,***p value < 0.001.

To find out the critical DUBs that are potentially involved in OXA resistance in HCC, we first constructed an OXA‐resistant HCC cell line (Huh‐7/OXA). We measured the survival percentage of HCC cells treated with OXA. Results showed that Huh‐7/OXA cells had higher survival rate compared with the parental Huh‐7 cells under the treatment of OXA (Figures 1A–C). We then performed RNA‐seq analysis of Huh‐7 and its OXA‐resistant counterpart (Huh‐7/OXA). The expression of DUBs was shown in Figure 1D. KEGG pathway analysis was performed to find out the OXA resistance‐related pathways in HCC. Consistent with previous findings, our results demonstrated that the antioxidant stress pathway, Wnt signaling pathway and p53 signaling pathway were dysregulated in Huh‐7/OXA cells (Figure 1E), these pathways have been previously identified to be associated with drug resistance. And we also found that the GSH metabolism pathway and ferroptosis pathway were significantly enriched, which allows us to consider the role of ferroptosis in OXA resistance.^41^ We then performed RT‐PCR analysis to confirm the RNA‐seq results. As shown in Figure 1F, we observed that USP20 was the most significantly upregulated DUB in Huh‐7/OXA cells. Western blot analysis revealed that the protein level of USP20 was also markedly increased in Huh‐7/OXA cells (Figure 1G). Next, we detected the expression of USP20 in HCC tissues and normal tissues through both immunohistochemical (IHC) and Western blotting methods. Our results indicated that USP20 was expressed at low levels in normal liver samples and at high levels in HCC clinical samples, especially in recurrent tumors (Figures 1H and I). Moreover, high USP20 expression in HCC patients indicated poor prognosis (Figure 1J). These results suggest that high expression of USP20 is correlated with OXA resistance and serves as an adverse prognostic biomarker for HCC patients.

### 2.2 USP20 promotes the OXA resistance, ferroptosis resistance, and stem‐like properties

**FIGURE 2:**
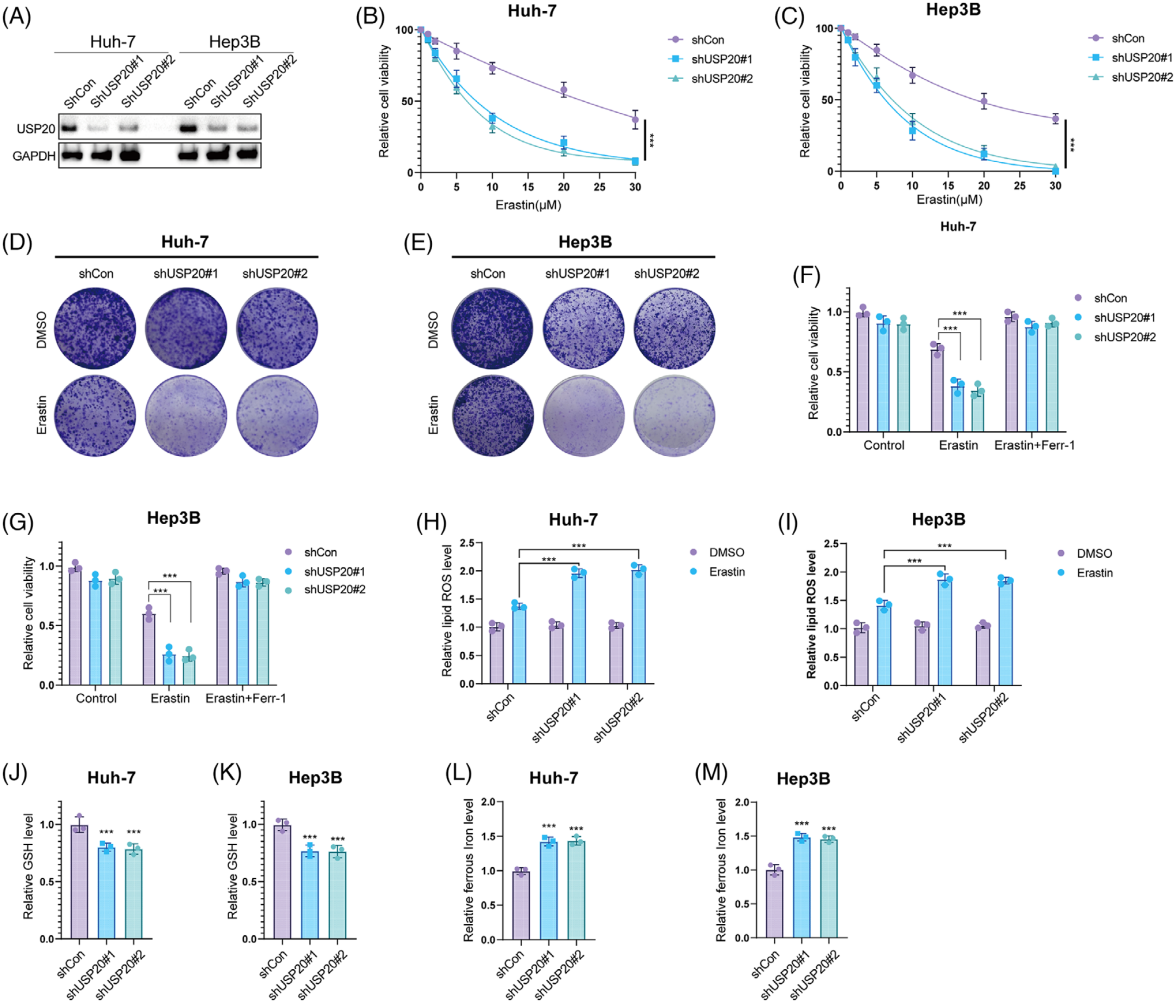
USP20 knockdown confers ferroptosis of HCC cells. (A) knockout efficiency of USP20. (B and C) The relative cell viability of Huh‐7 and Hep3B cells treated with the indicated dosage of erastin for 24 h. (Each group contained three replicates.) (D and E) Crystal violet staining of HCC cells treated with erastin. (F and G) CCK8 assay showing the response of HCC cell lines to erastin (20 μM) ± ferrostatin (1 μM) for 24 h. (H–M) Lipid ROS (H and I), GSH levels (J and K), and ferrous iron levels (L and M) were measured in HCC cells. Results shown are representative of three independent experiments. Data are represented as mean ± SD of biological triplicates.*p value < 0.05**, *p* value < 0.01, ***p value < 0.001.

**FIGURE 3:**
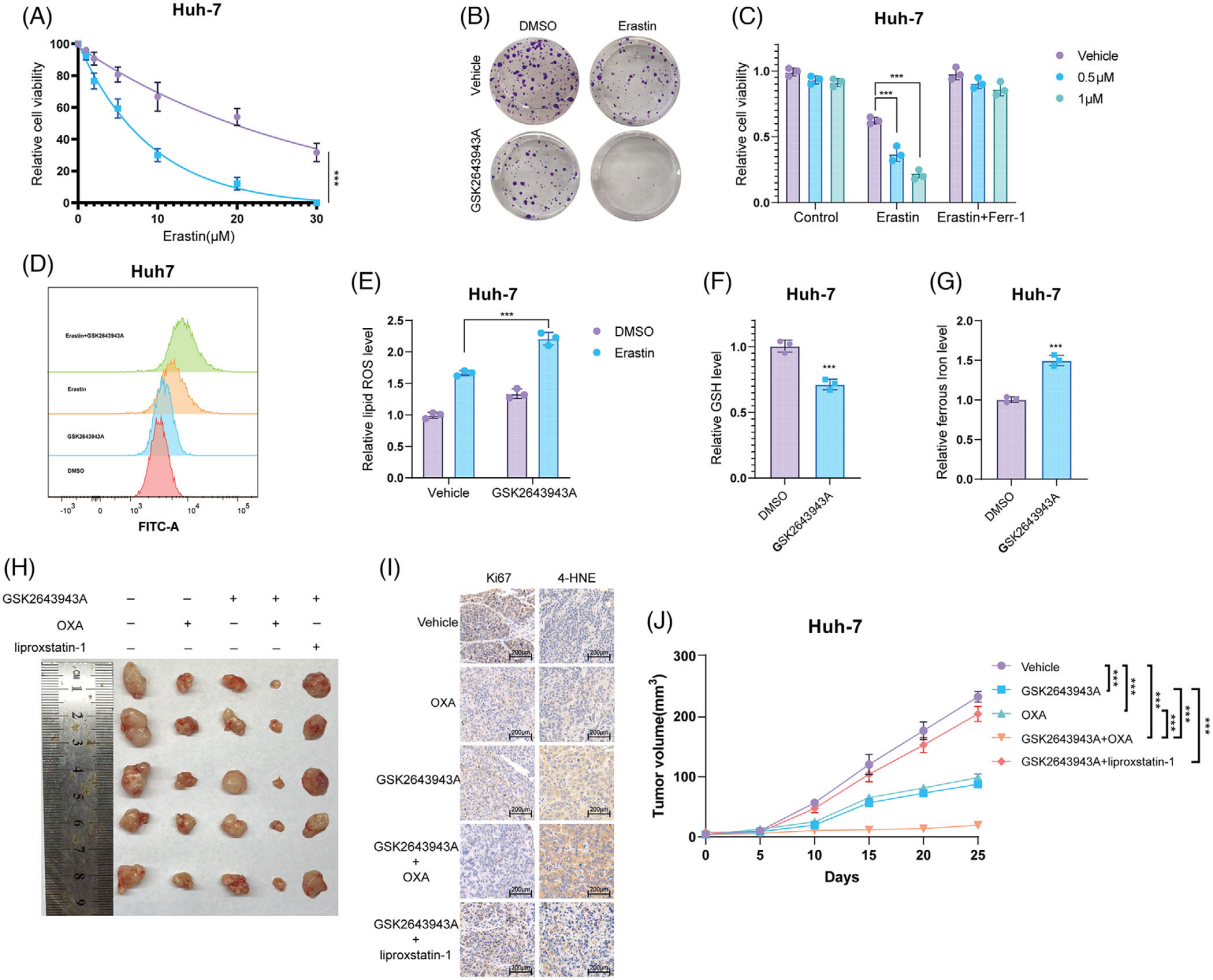
Inhibition USP20 by GSK2643943A promotes ferroptosis of HCC cells. (A) The relative cell viability of Huh‐7 cells treated with the indicated dosage of erastin for 24 h. (Each group contained three replicates.) (B) Crystal violet staining of HCC cells treated with erastin. (C) CCK8 assay showing the response of HCC cell lines to erastin (20 μM) ± ferrostatin (1 μM) for 24 h. (D–G) Lipid ROS (D and E), GSH levels (F), and ferrous iron levels (G) were measured in HCC cells. (H–J) GSK2643943A treatment inhibited the tumor growth in vivo. 1 × 106 Huh‐7 cells were injected to the right dorsal flank of each mouse (*n* = 6). After the tumors reached approximately 50 mm^3^, mice were treated with OXA (5 mg/kg, twice a week). The animals were then treated with the vehicle or GSK2643943A intraperitoneally (30 mg/kg/day) with or without liproxstatin‐1 (10 mg/kg/day, intraperitoneally). Tumor sizes were measured every 5 days until the end of the experiment. Results shown are representative of three independent experiments. Data are represented as mean ± SD of biological triplicates.*, *p* value < 0.05, **p value < 0.01, ***p value < 0.001.

We then investigated the biological functions of USP20 using two nonoverlapping shRNAs in HCC cells. Huh‐7, Hep3B cells, and HCC primary cell line (from sample T1 with high USP20 expression) were treated with USP20 shRNAs and the viability of HCC cells was measured using CCK8. As shown in Figures S1A and S1B, knockdown of USP20 with specific silencers significantly increased the sensitivity of the Huh‐7 and Hep3B to OXA. Consistently, Calcein AM/PI staining and crystal violet assay indicated that the combination of USP20 silence and OXA (10 μM, approximately induced half of HCC cells death) leads to increased cell death (Figures S1C–E). The same results were observed in HCC primary cells (Figures S2A–C). We utilized a xenograft model to assess the role of USP20 in HCC in vivo. It is found that knockdown of USP20 significantly reduced tumor growth and increased the antitumor efficacy of OXA (5 mg/kg, twice a week)^20^ in vivo (Figure S2D). As ferroptosis pathway were enriched in OXA‐resistant cells, we further examined the functions of USP20 in regulating HCC ferroptosis. Huh‐7, Hep3B, and HCC primary cell line were treated with increasing concentration of a glutamate–cystine antiporter system Xc− inhibitor (erastin), and found that USP20 depletion inhibited the viability of HCC cells, and cells depleted with USP20 were more sensitive to ferroptosis induced by erastin treatment (Figures 2A–C and S2E). As determined by the crystal violet staining assays, shRNA‐mediated ablation of USP20 elevates cell sensitivity to erastin (Figures 2D, E and S2F). We then detected erastin‐induced ferroptosis (20 μM, approximately induced half of HCC cells death) with or without ferrostatin‐1 (Ferr‐1; 1 μM),^42^ a ferroptosis inhibitor. We observed that USP20 depletion reduced the cell viability upon the treatment of erastin, which could be rescued by Ferr‐1, suggesting that the mechanism is specific in cell ferroptosis (Figures 2F, G and S2G). Moreover, we used RSL‐3 (ferroptosis activator) to further confirm our results identified above. It is found that USP20 depletion increased cell sensitivity to RSL‐3 treatment, which could be blocked by the inhibitors of ferroptosis, such as Ferr‐1, and liproxstatin‐1, but not by the necroptosis inhibitor necrosulfonamide, apoptosis inhibitor Z‐VAD‐FMK, or autophagy inhibitor 3‐methyladenine (Figures S3A and B). It is also found that USP20 depletion decreased the GSH levels in HCC cells, while increased lipid ROS levels and ferrous iron levels (Figures 2H–M and S2H–J). We also tested the effect of pharmacological USP20 inhibitor GSK2643943A (1 μM)^43^ on HCC cells. It is found that GSK2643943A significantly increased cell sensitivity to OXA (Figures S4A–C). And GSK2643943A treatment markedly reduced the proliferation of Huh‐7 cells (Figure S4D). Consistent with the USP20‐knockdown results, GSK2643943A decreased the cell viability and increased erastin‐induced viability inhibition and increased ferrous iron levels and lipid ROS levels, while decreased the GSH levels in Huh‐7 cells (Figures 3A–G). As expected, GSK2643943A increased cell sensitivity to RSL‐3 treatment, and such effect was blocked by Ferr‐1 and liproxstatin‐1 (inhibitors of ferroptosis) but not by the necroptosis inhibitor necrosulfonamide, the apoptosis inhibitor Z‐VAD‐FMK, or autophagy inhibitor 3‐methyladenine (Figures S3C and D). Furthermore, in vivo tumorigenesis assay indicated that GSK2643943A significantly reduced tumor growth. In addition, administration of GSK2643943A (30 mg/kg/day)^44^ and OXA (5 mg/kg, twice a week) in combination significantly decreased tumor growth than administration of either GSK2643943A or OXA alone. And liproxstatin‐1 (10 mg/kg/day, intraperitoneally)^45^ could reverse the suppression effect induced by GSK2643943A. 4‐HNE staining result showed that GSK2643943A or GSK2643943A and OXA in combination markedly induced 4‐HNE production, while OXA treatment alone did not affect the production of 4‐HNE (Figures 3H–J). These results indicated that inhibition of USP20 by GSK2643943A could induce ferroptosis of HCC in vivo. Previous studies revealed that cancer stem cells (CSCs) were responsible for tumor chemotherapy resistance, metastasis, and recurrence,^46^ we then investigated the functions of USP20 in HCC stemness. We observed that USP20 depletion remarkably inhibited the oncosphere formation of Huh‐7, Hep3B and HCC primary cells (Figures S2K, S5A, andB). In addition, depletion of USP20 significantly decreased the expression of pluripotent transcription factors Nanog, Sox2, c‐Myc, and Oct4 (Figures S5C and D). Moreover, HCC cells expressing shUSP20 displayed an inhibited tumor‐initiation capacity in NOD‐SCID mice (Figures S5E and F). Overall, USP20 is associated with the OXA resistance, ferroptosis, and stem‐like properties.

### 2.3 USP20 stabilizes SLC7A11 through the deubiquitylation activity

**FIGURE 4:**
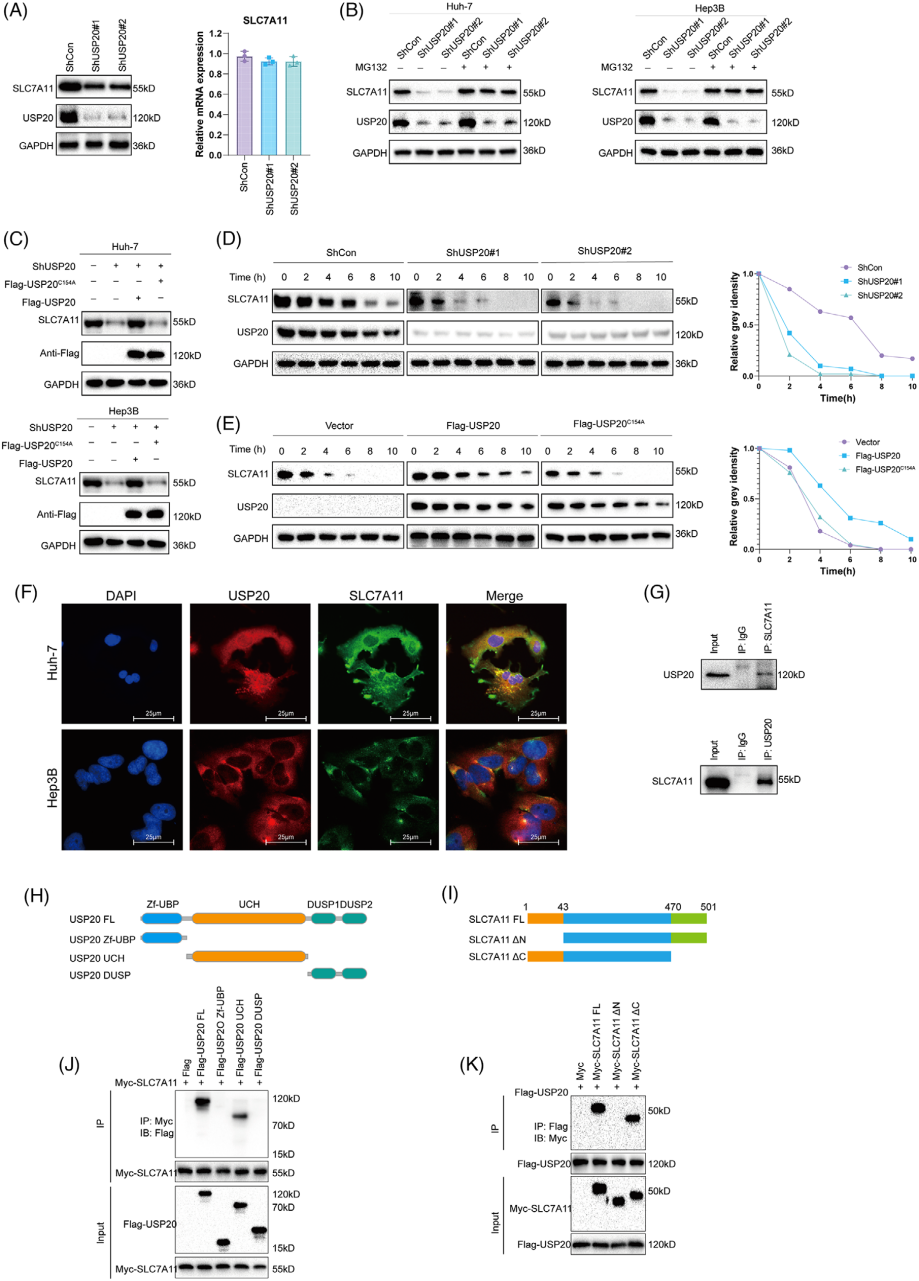
USP20 associates with SLC7A11 and increases its stability. (A) USP20 depletion decreased SLC7A11 protein level without affecting mRNA expression of SLC7A11. (B) HCC cells transfected with the indicated shRNA were treated with or without the proteasome inhibitor MG132 (10 μM, 6 h), and then proteins were analyzed. (C) USP20 WT or C154A was introduced into HCC cells together with USP20 shRNA. SLC7A11 levels were measured. (D) Huh‐7 cells transfected with USP20 shRNA were treated with cycloheximide (10 μg mL^−1^), and collected at the indicated times for western blot. Quantification of β‐catenin levels relative to GAPDH is shown. (E) Half‐life analysis of SLC7A11 in HEK293 cells transfected with the indicated plasmids. (F) An immunofluorescence assay demonstrated that USP20 andSLC7A11 at least partially colocalized in Huh‐7 and Hep3B cells. (G) Co‐IP assay reveals association between endogenous USP20 and SLC7A11 in Huh‐7 cells. Huh‐7 cells were harvested with RIPA lysis buffer. Co‐IP was performed using antibody as indicated. (H and I) USP20 and SLC7A11 domain structure and deletion mutants used in the study. (J) The UCH domain of USP20 interacted with SLC7A11. HEK293 cells were transfected with 2 μg Myc‐SLC7A11 together with Flag‐USP20 full length or mutants. After 24 h, cells were harvested with NP‐40 lysis buffer. Co‐IP was performed using Myc antibody. The possible interacted USP20 domains were detected by Flag antibody. (K) The N terminal of SLC7A11 interacted with USP20. HEK293 cells were transfected with 2 μg Flag‐USP20 together with Myc‐SLC7A11 full length or mutants. After 24 h, cells were harvested with NP‐40 lysis buffer. Co‐IP was performed using Flag antibody. The possible interacted β‐catenin domains were detected by Myc antibody.

SLC7A11 is regarded as a key regulator of ferroptosis which can import cystine for the biosynthesis of GSH and antioxidant defense,^34^, ^47^ we went on to examine the expression of SLC7A11 in HCC cells. As expected, USP20 depletion remarkably reduced the protein levels of SLC7A11 without influence on the mRNA level (Figure 4A). Since USP20 is a DUB belonging to the ubiquitin‐specific processing protease family and USP20 depletion decreased SLC7A11 protein levels, suggesting that USP20 may control the protein stability of SLC7A11 via the Ub–proteasome system. We observed that USP20 depletion significantly reduced the protein expression of SLC7A11, and such effect could be blocked by the addition of MG132 (proteasome inhibitor) or overexpression of wild‐type USP20, but not its catalytically inactive mutant (Figures 4B and C). To prove that USP20 affects SLC7A11 stability, we treated Huh‐7 cells with the protein synthesis inhibitor cycloheximide (CHX). SLC7A11 stability was significantly reduced in cells depleted with USP20, while increased in cells overexpressing USP20‐WT but not USP20‐C154A (Figures 4D and E). These results suggested that USP20 enhanced SLC7A11 protein stability through the Ub–proteasome system. Immunofluorescence assay was carried out to detect the cellular localization of USP20 and SLC7A11. Immunostaining results suggested that USP20 and SLC7A11 both localized in the cytosol of Huh7 and Hep3B cells (Figure 4F). Further coimmunoprecipitation (Co‐IP) results revealed that endogenous USP20 could coimmunoprecipitate with endogenous SLC7A11 (Figure 4G). In addition, deletion analysis indicated that the N terminal of SLC7A11 could physically bind to the UCH domain of USP20 (Figures 4H–K).

**FIGURE 5:**
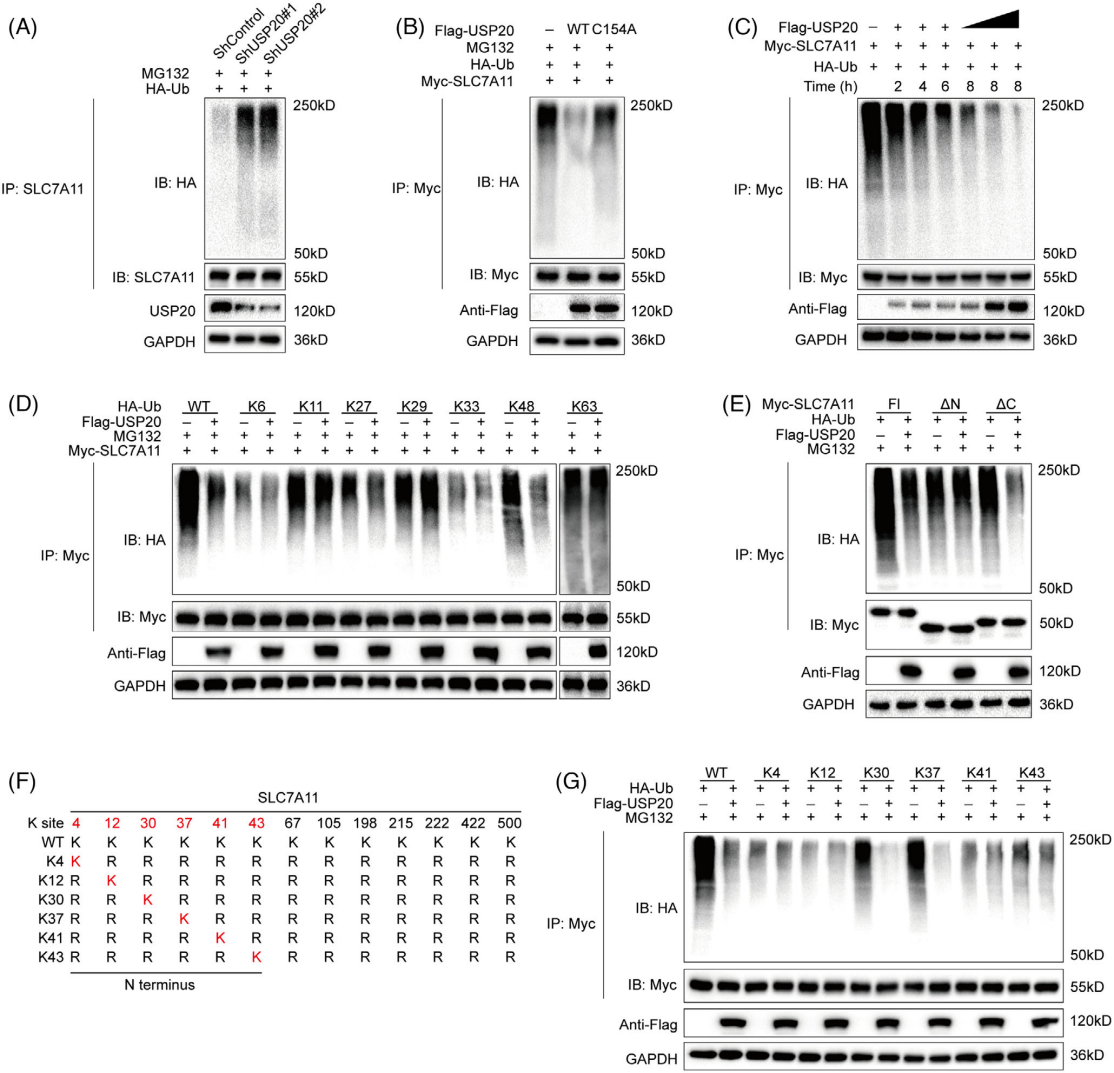
USP20 de‐polyubiquitylates SLC7A11. (A) Huh‐7 cells transfected with the indicated shRNA were treated with MG132 for 6 h before collection. SLC7A11 was immunoprecipitated with anti‐SLC7A11 and immunoblotted with anti‐HA. (B) Immunoblotting was used to detect the ubiquitination of SLC7A11 in HEK293T cells cotransfected with Myc‐SLC7A11, HA‐ubiquitin and Flag‐USP20 (wild type or C154A). (C) USP20 removed the ubiquitin chain of SLC7A11 in a time‐ and dose‐dependent manner. (D) HA‐WT, K6, K11, K27, K29, K33, K48, or K63 Ub were cotransfected with Myc‐SLC7A11 and Flag‐USP20 into HEK293T cells. After treatment with 10 μM MG132 for 6 h, cell lysates were subjected to ubiquitination assay and the ubiquitination level of SLC7A11 detected by HA antibody. (E). Immunoblotting to detect the ubiquitination of the SLC7A11 deletion mutants (FL, ΔN, and ΔN) in HEK293T cells cotransfected with Myc‐SLC7A11 (FL, ΔN, and ΔN), HA‐Ub and USP20. (F) A schematic diagram of SLC7A11 and its mutants. (G) Immunoblotting to detect the ubiquitination of SLC7A11 mutants (K4, K12, K30, K37, K41, and K43) in HEK293T cells cotransfected with Myc‐SLC7A11 mutants, USP20 and HA‐Ub.

We further investigated whether SLC7A11 is a substrate of USP20. As shown in Figure 5A, the ubiquitination of SLC7A11 was markedly increased upon USP20 knockdown. Conversely, ectopic expression of USP20‐WT significantly decreased ubiquitinated SLC7A11 in cells (Figure 5B). In vivo ubiquitination assays showed that USP20 decreased SLC7A11 ubiquitination in a time‐ and dose‐dependent manner (Figure 5C). We also performed ubiquitination assay using a series of mutant ubiquitin to investigated which kind of ubiquitination of SLC7A11 was hydrolyzed by USP20, including K6, K11, K27, K29, K33, K48, and K63. It is found that USP20 could selectively cleave K48‐linked ubiquitin chains from USP20 (Figure 5D). To further examine which region of SLC7A11 was regulated by USP20, we carried out a ubiquitination assay using the ΔN‐terminal, ΔC‐terminal, or full‐length plasmids of SLC7A11 and USP20. We found that USP20 significantly hydrolyzed the ubiquitin chains on the N terminus of SLC7A11, but displayed no influence on the C terminus of SLC7A11 (Figure 5E). We then predicted the potential ubiquitination sites of SLC7A11 protein using the online bioinformatic tools (UbPred, UbiSite, and BDM‐PUB), a total of 13 possible ubiquitination sites were identified in the full‐length of SLC7A11 protein, including six ubiquitination sites located at the N terminus. We further mutated the lysine residues of SLC7A11 to find out the specific sites that are deubiquitinated by USP20 (Figure 5F). Ubiquitination assay indicated that K30 and K37 were the key sites on SLC7A11 deubiquitinated by USP20 (Figure 5G). To determine the role of USP20 catalytical activity in HCC, we overexpressed USP20 WT, and catalytically inactive mutant C154A in HCC primary cell line (from sample T7 with low USP20 expression). Overexpression the wild type USP20 decreased the sensitivity of the HCC primary cell line to OXA, while USP20 C154A almost had no effect (Figures S6A–C). We examined the consequences of USP20 mutants on tumor growth, and found that only USP20 (WT) promoted xenograft growth (Figure S6D). Consistent with the results identified above, USP20 (WT) reduced cell sensitivity to erastin and increased the GSH levels, while decreased ferrous iron levels and lipid ROS levels of HCC cells (Figures S6E and I). The wild type USP20 enhanced the oncosphere formation of HCC cells. However, USP20 C154A almost had no such effects (Figure S6J). These results suggested that USP20 may act as a SLC7A11‐directed DUB which deubiquitylates and stabilizes SLC7A11.

### 2.4 ATR phosphorylates and stabilizes USP20

**FIGURE 6:**
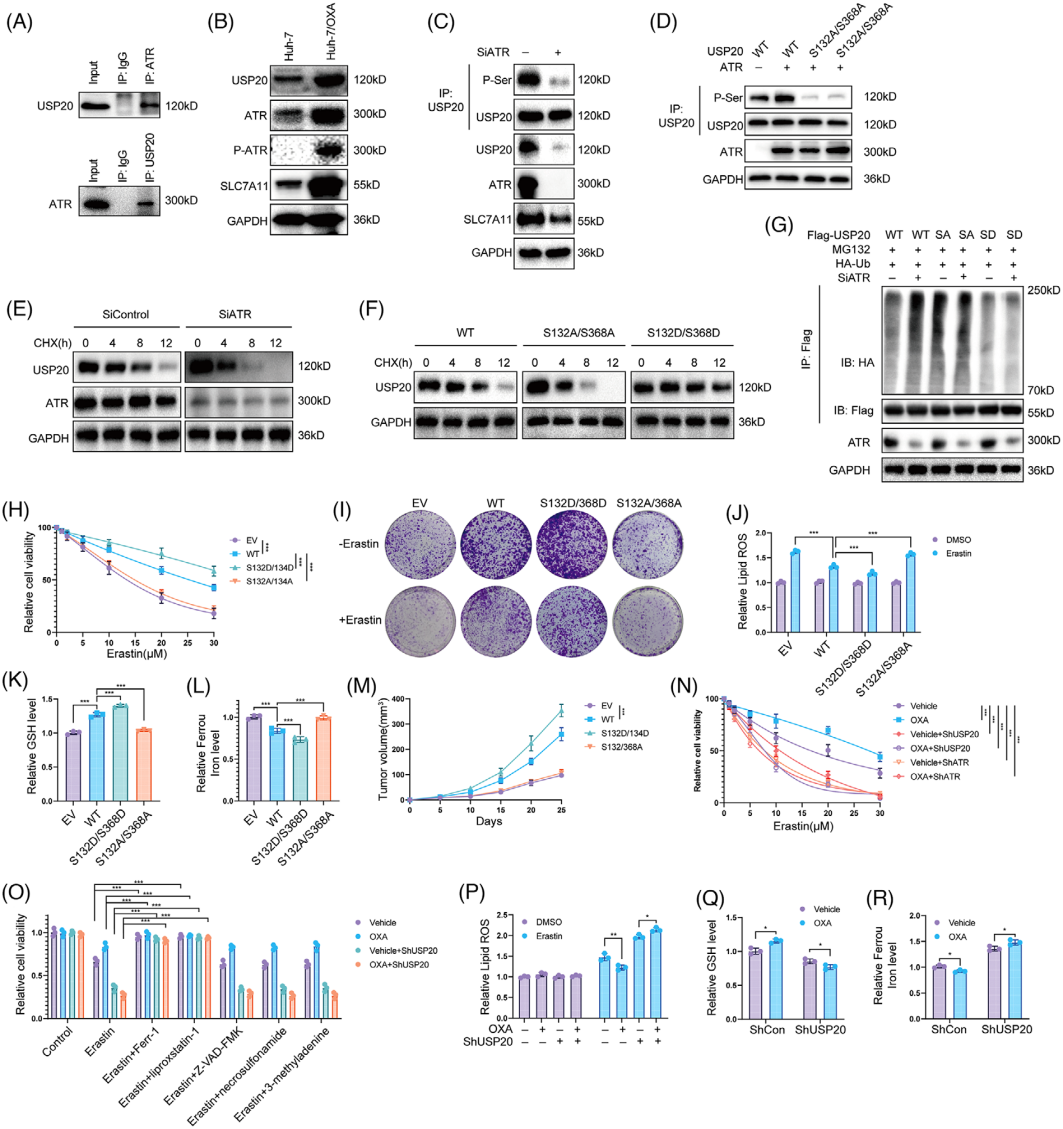
ATR phosphorylates USP20 and induces USP20‐dependent ferroptosis resistance. (A) Co‐IP assay reveals association between endogenous USP20 and ATR in Huh‐7 cells. Huh‐7 cells were harvested with RIPA lysis buffer. Co‐IP was performed using antibody as indicated. (B) The expression of ATR, P‐ATR, USP20, and SLC7A11 in Huh‐7/OXA and its parental cells. (C) Coimmunoprecipitation analysis of USP20 serine phosphorylation in Huh‐7 cells treated with ATR siRNA or siControl. (D) ATR phosphorylates S132 and S368 sites of USP20. (E) Inhibition of ATR by siRNA affected the turnover of USP20. Huh‐7 cells were treated siATR. Cells were then treated with CHX for the indicated time, and the expression of USP20 was analyzed by western blotting. (F) USP20 phosphorylation prolonged its half‐life. (G) HEK293T cells transfected with HA‐ubiquitin and Flag‐USP20 or indicated Flag‐USP20 mutant plasmids were treated ATR siRNA. After treatment with 10 μM MG132 for 6 h, cell lysates were subjected to ubiquitination assay and the ubiquitination level of USP20 was detected by HA antibody. (H) The relative cell viability of HCC primary cells treated with the indicated dosage of erastin. (I) Crystal violet staining of HCC primary cells treated with erastin. (Each group contained three replicates.) (J–L) Lipid ROS (J), GSH levels (K), and ferrous iron levels (L) were measured in HCC primary cells. (M) In vivo xenografts generated from primary HCC cells expressing an empty vector, USP20‐WT, USP20‐S132A/S368A, or USP20‐ S132D/S368D. 1 × 10^6^ primary HCC cells were injected to the right dorsal flank of each mouse (*n* = 6). Tumor sizes were measured every 5 days until the end of the experiment. (N) Huh‐7 cells transfected with shUSP20 or shControl plasmids were treated with OXA for 12 h. Then Huh‐7 cells were treated with increasing concentration of erastin for 24 h. The relative cell viability of HCC cells was detected using CCK8 assay. (Each group contained three replicates.) (O) Huh‐7 cells transfected with shUSP20 or shControl plasmids. CCK8 assay showing the response of HCC cells were treated with erastin (20 μM) or RSL‐3 (5 μM) in the absence or presence of ferrostatin‐1 (1 μM), liproxstatin‐1 (1 μM), Z‐VAD‐FMK (10 μM), necrosulfonamide (0.5 μM), or 3‐methyladenine (250 μM) for 24 h. (P–R) Huh‐7 cells transfected with shUSP20 or shControl plasmids. Lipid ROS (P), GSH levels (Q) and ferrous iron levels (R) were measured in HCC cells. Results shown are representative of three independent experiments. Data are represented as mean ± SD of biological triplicates.*p value < 0.05, **p value < 0.01, ***p value < 0.001.

Recent studies reported that the posttranslational modifications of DUBs can influence their stability and deubiquitinase activity.^48^, ^49^, ^50^ We then ectopic expressed Flag‐USP20 in HCC cells and performed immunoprecipitation (IP)‐based MS. Phosphorylation of USP20 at Ser132 and Ser368 residues was detected through MS analysis (Figure S7A). Interestingly, we discovered that ATR, a key player in replication stress signaling,^51^ was copurified with Flag‐USP20 (Figure S7B). We conducted Co‐IP analysis in Huh‐7 cell lines and observed the interaction between ATR and USP20 (Figure 6A). We observed that OXA‐resistant Huh‐7 cells expressed high levels of ATR, P‐ATR, USP20, and SLC7A11 (Figure 6B). Inhibition of ATR by BAY1895344 or siRNA markedly decreased the phosphorylation of USP20 as well as its protein levels (Figures 6C and S7C). We then performed point mutation and IP to verify that ATR phosphorylates S132 and S368 sites of USP20. We generated the USP20 mutant vector (USP20‐S132A/S368A) by replacing serine with alanine, and our results indicated that ATR phosphorylates wild‐type USP20 (USP20‐WT) but not USP20‐S132A/S368A (Figure 6D). We then examined whether ATR‐mediated USP20 phosphorylation affected its stabilization. Following CHX chase, we observed that suppression of ATR shortened USP20 half‐life (Figures 6E and S7D). Consistently, the phosphorylation‐null USP20 (S132A/368A) was destabilized while the phosphomimetic USP20 (S132D/S368D) mutant was stabilized (Figure 6F). The S132D/S368D mutant showed a decreased, whereas S132A/368A mutant showed an increased ubiquitination levels compared with the wild type USP20, and inhibition of ATR led to an increased ubiquitination of wild type but not mutant USP20 (Figures 6G and S7E). We then stably expressed USP20 WT, S132A/368A, and S132D/S368D in HCC primary cell line (from sample T7 with low USP20 expression) to examine the functions of USP20 phosphorylation in HCC cells,. Overexpression the wild type and S132D/S368D of USP20 decreased the sensitivity of the HCC primary cell line to OXA, while USP20 S132A/368A almost had no effect (Figure S8). Consistent with the results identified above, USP20 WT and S132D/S368D reduced cell sensitivity to erastin and increased the GSH levels, while ferrous iron levels decreased and lipid ROS levels of HCC cells (Figures 6H–L). We examined the consequences of USP20 mutants on tumor growth, and found that USP20‐WT and S132D/S368D promoted xenograft growth (Figure 6M). As ATR phosphorylates USP20 and increases its protein levels, we further examined the effects of DNA‐damage induced ATR activation on ferroptosis. To exclude the influence of apoptosis induced by OXA, we treated HCC cells with OXA for 12 h to induce DNA damage response. OXA treatment (12 h) significantly decreased cell sensitivity to erastin and RSL‐3, and we found that knockdown of USP20 or ATR inhibits the decreased sensitivity to ferroptosis by OXA (Figures 6N and S9A). In addition, the effect induced by OXA was significantly inhibited by the inhibitor of ferroptosis (liproxstatin‐1 and Ferr‐1) but not by the necroptosis inhibitor necrosulfonamide, the apoptosis inhibitor Z‐VAD‐FMK, or autophagy inhibitor 3‐methyladenine (Figures 6O and S9B), indicating that OXA induced DNA damage increases the resistance of HCC cells to cell death by selectively enhancing their sensitivity to ferroptosis. In addition, lipid ROS levels and ferrous iron levels were decreased under OXA treatment, and GSH levels were significantly increased. However, in cells depleted with USP20, such effect was not observed (Figures 6P–R), suggesting that USP20 is required for OXA‐induced ferroptosis resistance. These results suggest that OXA induced DNA damage leads to USP20/SLC7A11‐dependent ferroptosis resistance in HCC cells.

## 3 DISCUSSION

**FIGURE 7:**
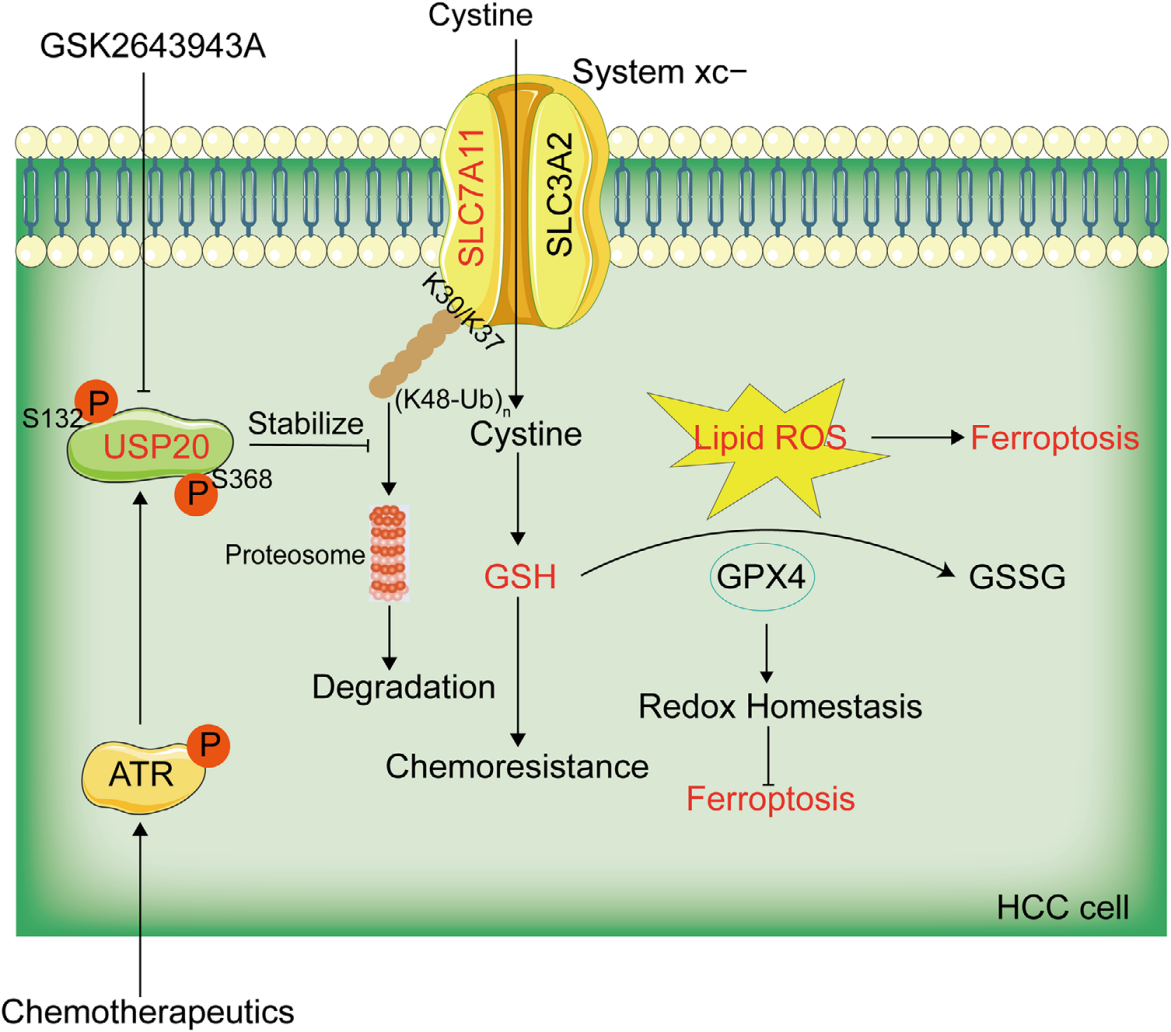
Graphic summary of ATR‐mediated activation of USP20/SLC7A11 pathway. USP20 stabilizes SLC7A11 via inhibiting K48‐specific polyubiquitination process on SLC7A11 protein at K30 and K37. DNA damage‐induced ATR activation is required for Ser132 and Ser368 phosphorylation of USP20. USP20 phosphorylation at Ser132 and Ser368 enhances its stability and thus confers OXA and ferroptosis resistance of HCC cells.

HCC is the most commonly diagnosed liver cancer and is an important medical problem. Despite decades of effort in understanding its cancerology, the potential molecule mechanisms that lead to the initiation and progression of HCC is complex and unclear. It is important to study the pathogenesis of HCC and identify more novel candidate targets for the clinical therapy. OXA has been approved as an effective systemic chemotherapeutic agent for the treatment of HCC, it displays better tolerability and improved efficacy compared to other parallel chemotherapeutic agents.^52^, ^53^ While the efficacy of chemotherapy is unsatisfactory, owing to chemoresistance in HCC, which becomes a major challenge encountered during the therapeutic treatment of HCC. Therefore, understanding the causes and mechanisms of drug resistance in HCC may improve the effects of chemotherapy on liver cancer, and thus, improve survival. A recent study revealed that in HCC patients, overexpression of PRMT3 may serve as a biomarker for OXA resistance. PRMT3 promotes OXA‐resistance through the methylation of IGF2BP1 at R452, which is essential for IGF2BP1 to stabilize the mRNA of HEG1.^9^ Growing evidence has demonstrated that targeting specific deubiquitinating enzymes (DUBs) is emerging as attractive strategy for cancer treatment. Therefore, we concentrated our study on DUBs involved in OXA resistance. In the present study, we observed that USP20 confers OXA and ferroptosis resistance of HCC cells. Mechanically, USP20 stabilized and deubiquitinated the antiferroptosis protein SLC7A11, which then protected cells from ferroptosis through the Xc−/GSH/GPX4 axis pathway. Most importantly, DNA damage‐induced ATR activation was required for Ser132 and Ser368 phosphorylation of USP20. USP20 phosphorylation at Ser132 and Ser368 enhanced its stability and thus conferred OXA and ferroptosis resistance of HCC cells (Figure 7). Pharmacological inhibition or knockdown of USP20 in HCC cells triggered ferroptosis and reversed OXA resistance both in vitro and in vivo. Our present study reveals a previously unrecognized link between OXA and ferroptosis and provides new insight into mechanisms regarding how DNA damage therapies always lead to therapeutic resistance.

Ferroptosis is a newly discovered form of iron‐dependent, programmed cell death induced by lipid peroxidation. Triggering ferroptosis is considered as an attractive method to circumvent therapy‐resistant cancer cells.^54^ Therapy‐resistant cancer cells with more property of epithelial–mesenchymal transition are more sensitive to GPX4 inhibition or statin treatment induced ferroptosis.^55^ In HCC, suppression the expression of SLC7A11 enabling HCC cells to overcome Sorafenib‐induced ferroptosis.^56^ Radiotherapy is reported to suppress the expression of SLC7A11, which enhances tumor lipid oxidation and sensitizes tumor cells to ferroptosis agonists in vitro and in vivo.^57^ A recent study reported that ionizing radiation (IR) induces reactive oxygen species and the expression of ACSL4, resulting in elevated lipid peroxidation and ferroptosis. As an adaptive response, IR also induces the expression of ferroptosis inhibitors, including SLC7A11 and GPX4, resulting radioresistance through inhibiting ferroptosis.^58^ These studies raise the intriguing questions of whether ferroptosis induction might enhance the therapeutic response to OXA treatments in HCC patients. Growing studies reported that DUBs play important roles in the progression and drug resistance of cancer. And DUBs inhibitors are supposed to be promising anticancer agents for cancer therapies. However, further investigations are needed to identify the potential mechanisms of DUBs and their inhibitors in the treatment of HCC.

USP20 is a member of DUB belonging to the ubiquitin‑specific protease (USP) family. Previous studies reported that USP20 regulates the deubiquitination of various target proteins to control different cellular events, such as HIF1α, TRAF6, type 2 iodothyronine deiodinase, sting/mita, β2 adrenergic receptor, SNAI2, and Claspin.^59^, ^60^, ^61^, ^62^, ^63^, ^64^ USP20 mediates ATR‐mediated DNA damage response through the stabilization of Claspin.^65^, ^66^ USP20 also regulates the hypoxia pathway by the deubiquitination of HIF1α.^67^ A recent study reported that USP20 stabilizes β‐catenin and activates Wnt/β‐catenin signaling pathway, thereby promoting the migration, invasion, and proliferation of cancer cells.^68^

In this study, we first constructed an OXA‐resistant HCC cell line and performed RNA‐seq analysis of Huh‐7 and its OXA‐resistant counterpart. We observed that USP20 was overexpressed in the OXA‐resistant HCC cells. High USP20 expression was correlated with poor prognosis in HCC patients. Knockdown of USP20 increased the sensitivity of the HCC cells to OXA. And the combination of OXA with USP20 silence leads to increased cell death than either USP20 silence or OXA alone. Animal studies demonstrated that USP20 depletion significantly inhibited tumor growth and increased the antitumor efficacy of OXA. KEGG analysis indicated that ferroptosis pathway was significantly enriched in OXA resistance cells, which allows us to consider the role USP20 ferroptosis. USP20 depletion also reduced the cell viability upon the treatment of erastin, which could be recovered by Ferr‐1, suggesting that the mechanism is specific in cell ferroptosis. We also assessed lipid ROS, GSH, and ferrous iron levels upon USP20 depletion. It was found that USP20 knockdown increased lipid ROS levels and ferrous iron levels, while decreased the GSH levels. We further examined GSK2643943A, a small molecular inhibitor of USP20, to verify our findings. Consistent with the results of USP20 depletion, GSK2643943A treatment significantly increased cell sensitivity to OXA and increased erastin‐induced viability inhibition. In vivo tumorigenesis assay indicated that administration of GSK2643943A and OXA in combination significantly inhibited tumor growth than administration of GSK2643943A or OXA alone. These results indicated that USP20 is an important regulator of OXA resistance and ferroptosis. We also observed that knockdown or pharmacologically inhibition of USP20 could significantly reduce the proliferation and viability of HCC cells, which was consistent with previous studies.^43^, ^64^, ^69^, ^70^, ^71^ SLC7A11 mediates the cystine/glutamate antiporter activity in the system Xc−, which plays a crucial role in ferroptosis. The protein stability and turnover of SLC7A11 could be controlled by several ubiquitination manners. SOCS2 promoted ferroptosis and radio‐sensitization of HCC cells. SOCS2 served as a bridge to transfer the attached ubiquitin to SLC7A11 which induced the degradation of SLC7A11 through K48‐linked polyubiquitination.^72^ OTUB1 could directly interact with and stabilize SLC7A11, leading to ferroptosis resistance.^73^ In this study, we further identified USP20 as a potential DUB responsible for SLC7A11, which deubiquitinated and stabilized SLC7A11 in HCC. First, USP20 and SLC7A11 binds with each other. Results of Co‐IP revealed the association between SLC7A11 and USP20. Further analysis indicated that the UCH domain of USP20 interacted with the N terminus of SLC7A11. Second, USP20 hydrolyzes the polyubiquitin chains of SLC7A11 and stabilizes SLC7A11 in a DUB activity‐dependent manner. USP20 knockdown markedly reduced SLC7A11 protein levels, and overexpression of USP20‐WT or addition of the proteasome inhibitor MG132 could reverse this effect. Under the treatment of CHX, USP20 knockdown significantly shortened the half‐life time of SLC7A11 protein. Ectopic expression of USP20‐WT, but not USP20^C154A^, markedly reduced the ubiquitylation of SLC7A11. In vivo deubiquitylation assays showed that USP20 reduced SLC7A11 polyubiquitination in a time‐ and dose‐dependent manner. To Further analysis indicated that K30 and K37 are the key sites on SLC7A11 deubiquitinated by USP20. We found that USP20 obviously hydrolyzed K48‐linked polyubiquitin chains from SLC7A11. As K48 polyubiquitination generally leads to proteasomal degradation,^74^, ^75^ USP20 may enhance the stability of SLC7A11 by removing the K48‐linked polyubiquitination from SLC7A11 protein. IP assay indicated that USP20 could be phosphorylated by ATR. Phosphorylation is key posttranscriptional modification and is involved in various intracellular signaling transduction pathways. In our present study, we found that inhibition of ATR significantly reduced the phosphorylation of USP20 and further increased the polyubiquitination of USP20, resulting its destabilization. Further study indicated that DNA damage induced by OXA treatment significantly decreased cell sensitivity to erastin. However, in cells depleted with USP20, such effect was not observed, suggesting that OXA induced DNA damage leads to USP20/SLC7A11‐dependent ferroptosis resistance in HCC cells. However, there were certain deficiencies somewhere in our study.  It is necessary to explore the correlations between OXA resistance and USP20 expression in HCC patients. Another, phosphor‐specific antibodies of USP20 S132 and S368 would make our results more credible.

In conclusion, our present work identified USP20 as a contributor to OXA resistance and suppress ferroptosis in HCC. Mechanically, USP20 deubiquitinated and stabilized the antiferroptosis protein SLC7A11 via hydrolyzing the K48‐linked ubiquitin chain, which then protected cells from ferroptosis. Most importantly, DNA damage‐induced ATR activation is required for Ser132 and Ser368 phosphorylation of USP20. USP20 phosphorylation at Ser132 and Ser368 enhances its stability and thus confers OXA and ferroptosis resistance of HCC cells. These results provide new insight into mechanisms regarding how DNA damage therapies always lead to therapeutic resistance. Therefore, targeting USP20 may mitigate the development of drug resistance and promote ferroptosis of HCC in patients receiving chemotherapy.

## 4 METHODS

### 4.1 Cell culture

The human embryonic kidney HEK293T, human Liver cancer cell lines Huh‐7 and Hep3B cells were purchased from the Procell Life Science&Technology Co, Ltd. All cell lines used in this study were authenticated with short tandem repeat analysis. HEK293T, Huh‐7, and Hep3B were cultured in Dulbecco's modified Eagle's medium (DMEM; Life Technologies, 41965) containing 10% fetal bovine serum (FBS; Gibco, Life Technologies, 10270). OXA‐resistant HCC cells were developed by exposing cells to OXA for 72 h at an intermittently increasing concentration. After 3 months, OXA‐resistant cells were harvested until the cells were stable in proliferation without significant death under the treatment of OXA.

HCC primary cells were prepared from specimens of fresh HCC samples which were immediately obtained after resection. The specimens were submerged into DMEM/F12 nutrient solution with antibiotic and cut into 1 mm^3^ with scissors. After rejection of necrotic tissue, dirty blood and blood vessels, the tissues were digested at 37°C with collagenase IV for 30 min. Next, the medium was passed through a cyto‐screen (*d* = 72 μm) and cells were collected through centrifugation at 300 g for 5 min. The cells were then treated with Red Blood Cell Lysis Buffer for red cell elimination and washed with PBS for three times. The HCC primary cells were enriched by centrifugation and inoculated into plastic Petri dishes. HCC primary cells were cultured in DMEM/F12 supplemented with 10% FBS. All cells were cultured at 37°C, in 5% CO_2_ humid atmosphere.

### 4.2 Plasmids and RNA inference

The wild type and mutant SLC7A11, USP20 plasmids were acquired from Hanbio Biotechnology Co., Ltd. The wild type and mutant Ub plasmids were obtained from Addgene. Small interfering RNAs targeting USP20 (shRNA‐1: 5′‐GCGACCATCATCAGGATCAAA‐3′; shRNA‐2: 5′‐ CCTATTGCTGTGGCTGATGAA‐3′) were synthesized by Hanbio Biotechnology Co., Ltd.

### 4.3 RNA extraction and qRT‐PCR analysis

Total RNA was extracted from samples using RNA Extraction Kit (Magen). RevertAid First Strand cDNA Synthesis Kit (Thermo) was used to perform reverse transcription according to the instructions. RT‐PCR analysis was performed using 7500 Fast Real‐Time PCR System (Applied Biosystems) with SYBR green mix (Vazyme) . Primers were listed as follows: USP20 (forward: 5′‐TGGGCTAGTCTGTAAGTCGC‐3′; reverse: 5′‐GGGACCTGCTCTTTGGATGTT‐3′); SLC7A11 (forward: 5′‐TGCTGGGCTGATT‐TATCTTCG‐3′; reverse: 5′‐GAAAGGGCAACCATGAAGAGG‐3′); c‐MYC (forward: 5′‐CCTTTGGGCGTTGGAAACC‐3′; reverse: 5′‐CGTCGCAGATGAAATAGGG‐3′); SOX2 (forward: 5′‐GCCCTGCAGTACAACTCCAT‐3′; reverse: 5′‐GACTTGACCACCGAACCCAT‐3′); Nanog (forward: 5′‐GTCCCAAAGGCAAACAACCC‐3′; reverse: 5′‐GCTGGGTGGAAGAGAACACA‐3′); Oct4 (forward: 5′‐TTTTGGTACCCCAGGCTATG‐3′; reverse: 5′‐GCAGGCACCTCAGTTTGAAT‐3′); GAPDH (forward: 5′‐ACGGGAAGCTTGTCATCAAT‐3′, reverse: 5′‐TGGACTCCACGACGTACTCA‐3′).

### 4.4 Co‐IP assay

Cells were harvested at 4°C using RIPA extraction reagent (Meilun) containing protease inhibitors (Meilun) after washed three times with prechilled PBS. Co‐IP assay was performed as we previously described.^76^ Each experiment was repeated in triplicate at least three times.

### 4.5 Protein stability assay

The half‐life of SLC7A11 was detected as we previously described.^76^ Each experiment was repeated in triplicate at least three times.

### 4.6 In vivo deubiquitination assay

We performed in vivo deubiquitination assay as described in our previous study.^76^ Briefly, HA‐Ub, Myc‐SLC7A11, Flag‐USP20, or Flag‐USP20^C154A^ plasmid were transfected into HEK293T cells for 48 h and treated with 10 μM MG132 (MCE) for another 6 h. Cells were harvested at 4°C using RIPA extraction reagent (Meilun) after washed three times with prechilled PBS. Ubiquitinated SLC7A11 was isolated and detected through Western blotting. In HCC cells, USP20 shRNAs and HA‐Ub plasmid were cotransfected into Huh‐7 cells. Ubiquitinated SLC7A11 was isolated and detected through Western blotting. Each experiment was repeated in triplicate at least three times.

### 4.7 Western blot analysis

Western blot analysis was performed as we previously described.^76^ The primary antibodies used for western blot analysis are listed as follows: SLC7A11 (Proteintech; 26864‐1‐AP; HUABIO, HA600098), USP20 (Proteintech; 17491‐1‐AP), GAPDH (Proteintech; 60004‐1‐Ig), Flag (Proteintech; 66008‐4‐Ig), Myc (Proteintech; 60003‐2‐Ig), HA (Proteintech; 51064‐2‐AP) antibodies. ECL (Meilun) and ChemiDocMP imager (Bio‐Rad) were used to detect and visualize the signals of bands.

### 4.8 Analysis of lipid ROS

Cells were harvested and incubated with 10 μM C11‐BODIPY (581/591) (#D3861; Thermo Fisher Scientific) in a cell culture incubator at 37°C for 30 min to measure the level lipid ROS. Then cells were collected and washed with PBS to remove excess C11‐BODIPY. Flow cytometer analysis was performed (Fortessa; BD Biosciences) with Texas Red channel and fluorescein isothiocyanate green channel. The experiment was repeated three times with three replicates per sample.

### 4.9 GSH assay

Total GSH was detected using the Glutathione Assay Kit (Beyotime Biotechnology) according to the instructions of manufacturer as we previously described.^77^ The experiment was repeated three times with three replicates per sample.

### 4.10 Iron assay

Iron assay kit (Sigma; Cat. MAK025) was used for the measurement intracellular ferrous (Fe2+) iron according to the instructions of manufacturer as we previously described.^77^ The experiment was repeated three times with three replicates per sample.

### 4.11 In vivo tumorigenesis assay

Four‐week‐old BALB/c nude mice were purchased from Beijing HFK Bioscience Co., Ltd. BALB/c nude mice were injected with 1 × 10^6^ HCC cells into the flanks subcutaneously at the age of 6 weeks (*n* = 6). Tumor sizes were measured and recorded every other day until the end of the experiment. Tumor volume  =  (max. diameter) × (min. diameter)^2^ × 0.5. All animal experiments were performed in accordance with the protocols approved by the ethnic committee of Xiangya Hospital.

### 4.12 Live/dead cell staining

HCC cells were incubated with 2 μM PI solution (Meiun) and 2 μM Calcein‐AM at 37°C for 15 min after washed three times with PBS. Cells were photographed using a fluorescence microscope (N2‐DMi8; Leica). The experiment was repeated three times with three replicates per sample.

### 4.13 Cell viability assay

CCK8 assay was used to determine cell viability as we previously described.^77^ The experiment was repeated three times with three replicates per sample.

### 4.14 Sphere formation assay

We performed sphere formation assay as previously reported by our group.^76^ Cells were plated into six‐well ultra‐low attachment plates (Corning) at a density of 2000 cells per well. Cells were cultured in serum‐free DMEM/F12 medium supplemented with 20 ng/mL bFGF, 20 ng/mL EGF, and B27 (1:50). After 14 days, the spheres were counted and photographed.

### 4.15 Limiting dilution assay in vivo

In vivo limiting dilution assay was performed as previously reported by our group.^76^ Briefly, spheroids were dissociated into single cells, NOD‐SCID mice were subcutaneously injected with indicated cells (5 × 10^3^, 1 × 10^4^, 5 × 10^4^, and 1 × 10^5^) into the flanks. Two months later, tumors were detected and the frequency of CSCs was determined using the extreme limiting dilution algorithm (http://bioinf.wehi.edu.au/software/elda/index.html).

### 4.16 IHC analysis

IHC analysis was performed as previously reported by our group.^78^ The liver cancer biopsies were acquired form the Department of Pathology at Xiangya Hospital and were validated by two pathologists independently. The research was approved by the Ethics Committee at Xiangya Hospital of Central South University (Approval number: 2022020074).

### 4.17 Statistical analysis

Statistical differences were calculated using two‐tailed student t tests, one‐way ANOVA with a Bonferroni's posttest, Kaplan–Meier with the log‐rank test where appropriate (GraphPad 7.0). Differences are considered statistically significant when *p* < 0.05. Data are represented as the mean ± SD.

## AUTHOR CONTRIBUTIONS

L. Z. and Y. T. designed and supervised the research. J. T., G. L., L. Z., D. X., S. L., L. X., and Y. T. performed research and provided helpful discussions. J. T., G. L., L. X., L. Z., D. X., S. L., and Y. T. analyzed and interpreted the data. D. X. conducted pathology evaluations. All authors reviewed and edited the manuscript. Y. T. contributed to all aspects of the study. G. L. performed animal model experiments. J. T. had a primary role in interpreting and organizing the data as well as writing the manuscript. All authors have read and approved the final manuscript.

## CONFLICT OF INTEREST STATEMENT

The authors declare no conflict of interest.

## ETHICS STATEMENT

The research was carried out according to the World Medical Association Declaration of Helsinki and was approved by the Ethics Committee at Xiangya Hospital of Central South University (Approval number: 2022020074).

## Supporting information

Supporting Information

Supporting Information

## Data Availability

The RNA sequence data are available at www.ncbi.nlm.nih.gov/bioproject/939902/. The data that support the findings of this study are available from the corresponding author upon reasonable request. Clinical information of patients was included in supplementary dataset.
