# Supplementary material for: ATR‐dependent ubiquitin‐specific protease 20 phosphorylation confers oxaliplatin and ferroptosis resistance
Source: MedComm (2020). 2023 Dec 20;4(6):e463. doi: 10.1002/mco2.463 (PMC10732327; doi:10.1002/mco2.463)
Supplement: Supplementary file 1 — Supporting Information [file MCO2-4-e463-s002.pdf]

ATR-dependent ubiquitin-specific protease 20 phosphorylation confers oxaliplatin and ferroptosis resistance

Jianing Tang[1,2], Guo Long[1], Desheng Xiao[3], Shuang Liu[4], Liang Xiao[1*], Ledu Zhou[1*], Yongguang Tao[5,6,7,8*]

1. Department of Liver Surgery, Xiangya Hospital, Central South University, Changsha, Hunan, 410078 China.
2. National Clinical Research Center for Geriatric Disorders, Xiangya Hospital, Central South University, Changsha, Hunan, 410008, China.
3. Department of Pathology, Xiangya Hospital, Central South University, Changsha, Hunan, 410078 China.
4. Department of Oncology, Institute of Medical Sciences, National Clinical Research Center for Geriatric Disorders, Xiangya Hospital, Central South University, Changsha, Hunan, China.
5. Department of Pathology, Key Laboratory of Carcinogenesis and Cancer Invasion (Ministry of Education), Xiangya Hospital, Central South University, Hunan, 410078 China.
6. NHC Key Laboratory of Carcinogenesis (Central South University), Cancer Research Institute and School of Basic Medicine, Central South University, Changsha, Hunan, 410078 China
7. Department of Thoracic Surgery, Hunan Key Laboratory of Early Diagnosis and Precision Therapy in Lung Cancer and Hunan Key Laboratory of Tumor Models and Individualized Medicine, Second Xiangya Hospital, Central South University, Changsha, 410011 China
8. Hunan Key Laboratory of Cancer Metabolism, Hunan Cancer Hospital and Affiliated Cancer Hospital of Xiangya School of Medicine, Central South University, Changsha, 410078, Hunan, China

Running title: USP20 stabilizes SLC7A11

***Correspondence:**

Prof. Liang Xiao, 110 Xiangya Road, 410078, Changsha, Hunan, China. E-mail: xiaoliangrick@csu.edu.cn

Prof. Ledu Zhou, 110 Xiangya Road, 410078, Changsha, Hunan, China. E-mail: zhould@csu.edu.cn

Pro. Yongguang Tao, 110 Xiangya Road, 410078, Changsha, Hunan, China. Phone: +8615787270206. E-mail: taoyong@csu.edu.cn

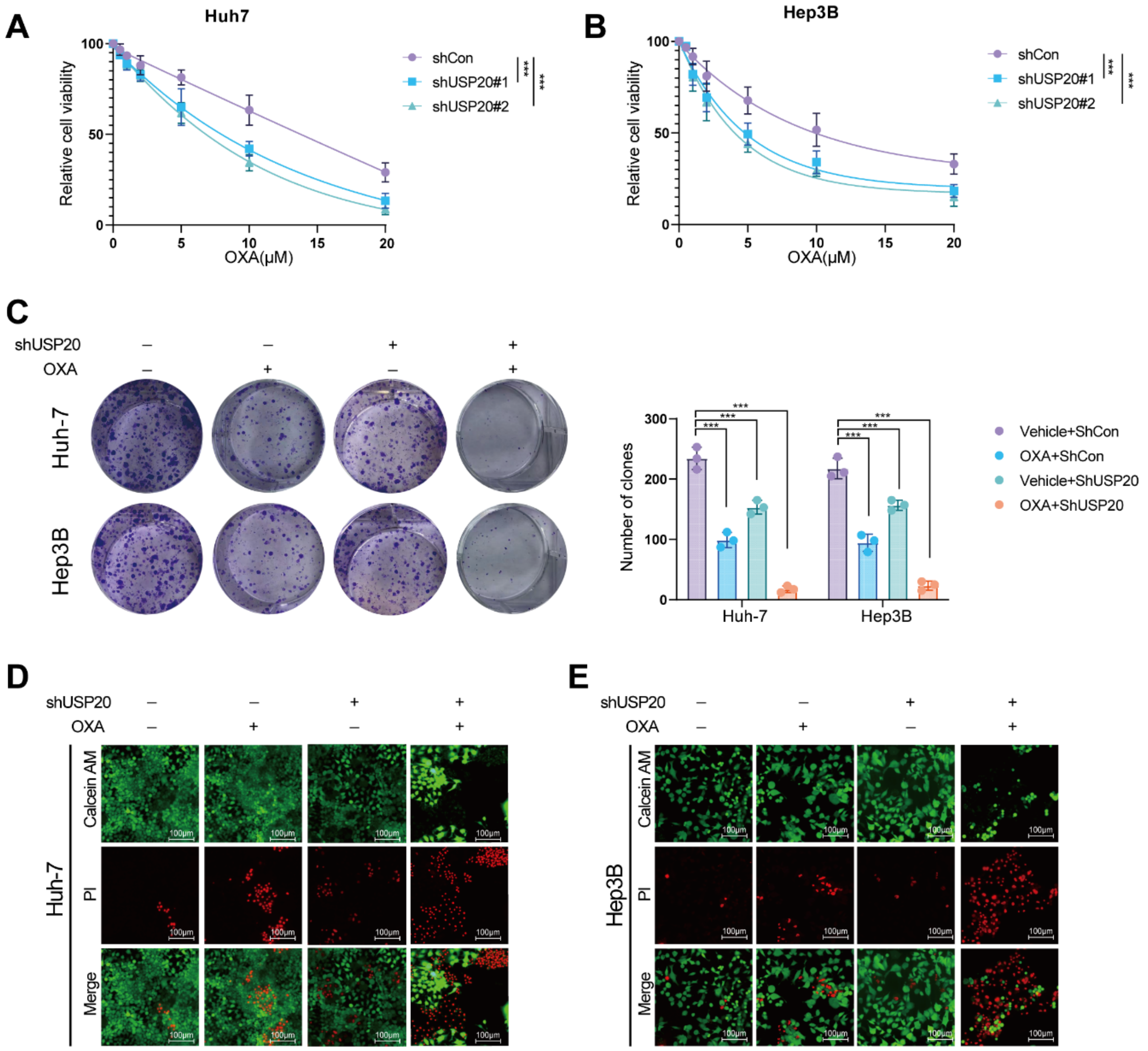

Figure S1. USP20 knockdown suppresses the OXA resistance of HCC cells. (A, B). The survival percentage of HCC cells treated with increasing concentration of OXA for 48h. (Each group contained 3 replicates). (C). Crystal violet staining of HCC cells treated with OXA. (D, E). Calcein/PI staining of HCC cells treated with OXA (10 μM) for 48h.

Results shown are representative of 3 independent experiments. Data are represented as mean ± SD of biological triplicates.*, *P value < 0.05; **, P value < 0.01; ***, P value < 0.001.*

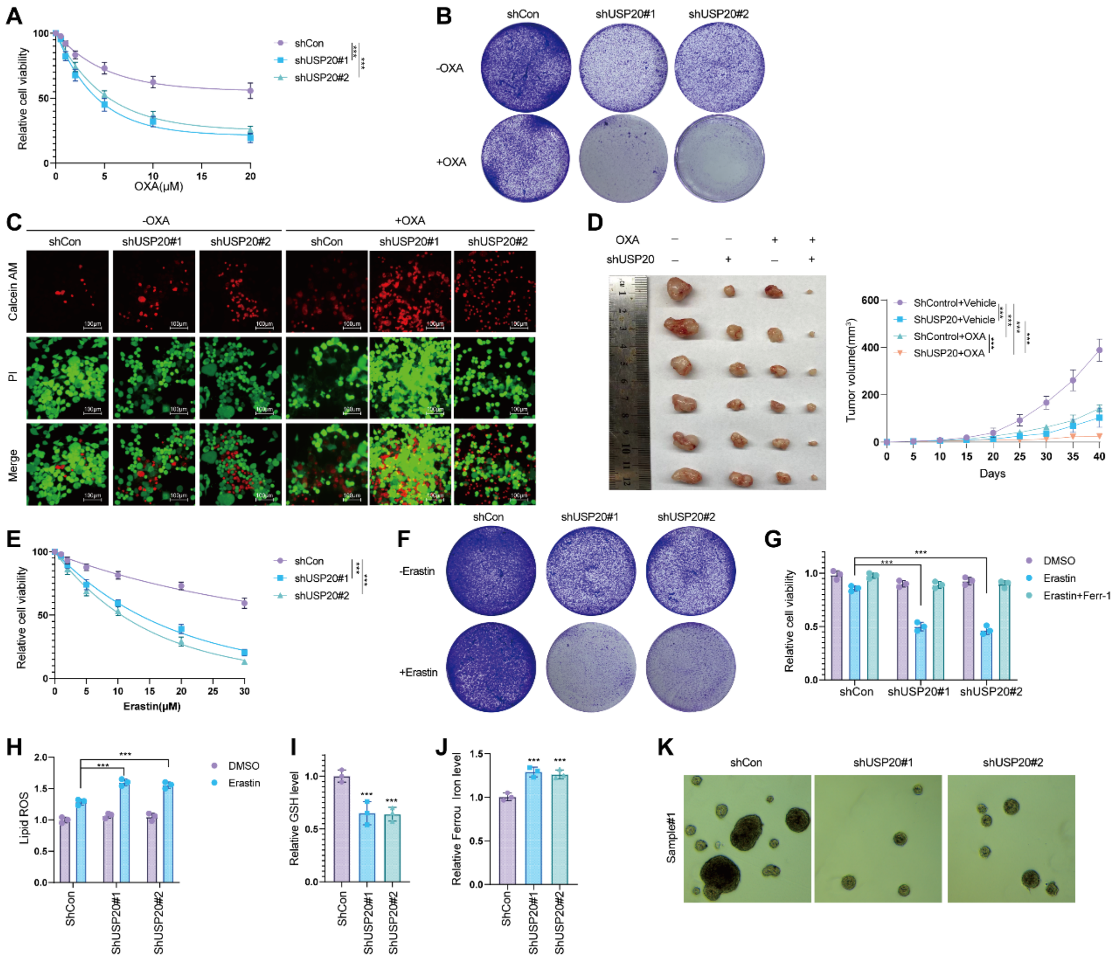

Figure S2. USP20 regulates the OXA resistance, ferroptosis and stem-like properties of primary HCC cells. (A). The survival percentage of primary HCC cells treated with increasing concentration of OXA for 48h. (Each group contained 3 replicates). (B). Crystal violet staining of primary HCC cells treated with OXA. (C). Calcein/PI staining of primary HCC cells treated with OXA (10 μM) for 48h. (D). In vivo xenografts generated from primary HCC cells expressing an empty vector or USP20-targeting shRNA and treated with OXA. 1×10^6 primary HCC cells were injected to the right dorsal flank of each mouse (n=6). After the tumors reached approximately 50 mm3, the mice were treated with OXA (5 mg/kg, twice a week). Tumor sizes were measured every 5 days until the end of the experiment. (E). The relative cell viability of primary HCC cells treated with the indicated dosage of erastin for 24h. (Each group contained 3 replicates). (F). Crystal violet staining of primary HCC cells treated with erastin. (G). CCK8 assay showing the response of primary HCC cells to erastin (20 μM)±ferrostatin (1 μM) for 24h. (H-J). Lipid ROS (H),

GSH levels (I) and ferrous iron levels (J) were measured in primary HCC cells. (K). Sphere formation assay of primary HCC cells.

Results shown are representative of 3 independent experiments. Data are represented as mean ± SD of biological triplicates.*, *P value < 0.05; **, P value < 0.01; ***, P value < 0.001.*

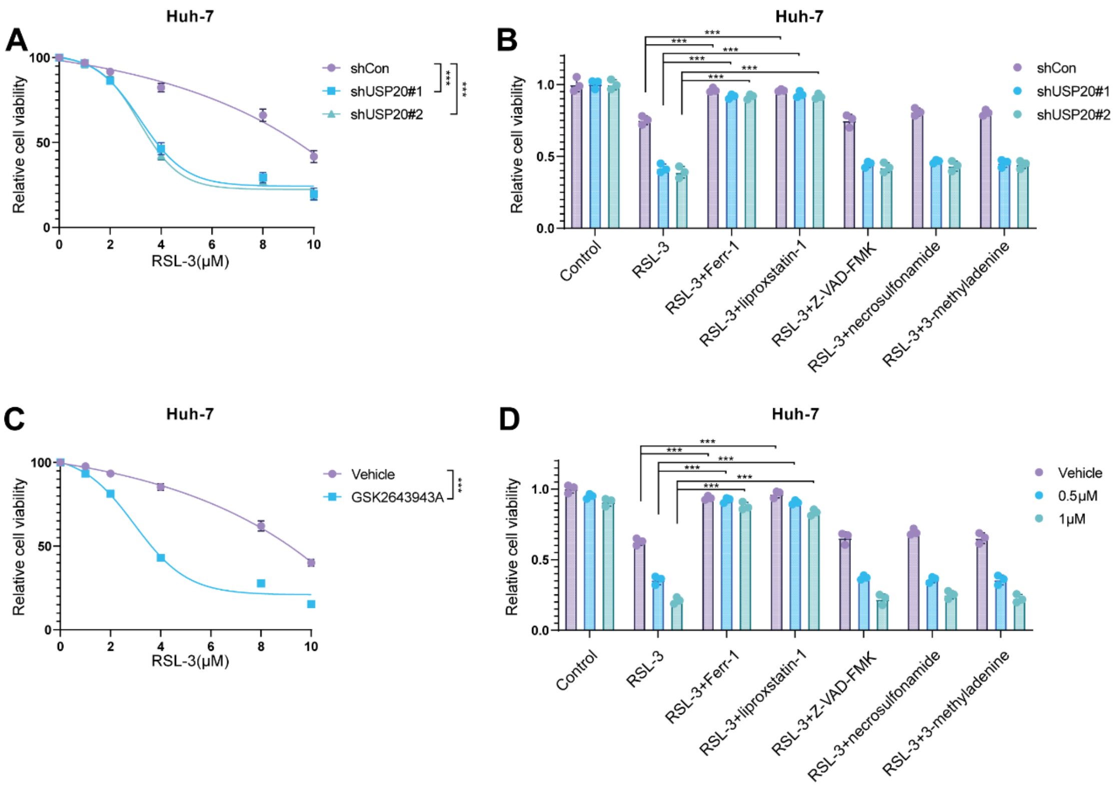

Figure S3. USP20 regulates the ferroptosis of HCC cells (A). The relative cell viability of primary HCC cells depleted with USP20 were treated with the indicated dosage of RSL-3 for 24h. (Each group contained 3 replicates). (B). CCK8 assay showing the response of HCC cells depleted with USP20 were treated with RSL-3 (5 μM) in the absence or presence of ferrostatin-1 (1 μM), liproxstatin-1 (1 μM), Z-VAD-FMK (10 μM), necrosulfonamide (0.5 μM), or 3-methyladenine (250 μM) for 24h. (C). The relative cell viability of primary HCC cells treated with GSK2643943A (1 μM) were treated with the indicated dosage of RSL-3 for 24h. (Each group contained 3 replicates). (D). CCK8 assay showing the response of HCC cells treated with GSK2643943A (1 μM) were treated with RSL-3 (5 μM) in the absence or presence of ferrostatin-1 (1 μM), liproxstatin-1 (1 μM), Z-VAD-FMK (10 μM), necrosulfonamide (0.5 μM), or 3-methyladenine (250 μM) for 24h.

Results shown are representative of 3 independent experiments. Data are represented as mean ± SD of biological triplicates.*, *P value < 0.05;* **, *P value < 0.01;* ***, *P value < 0.001.*

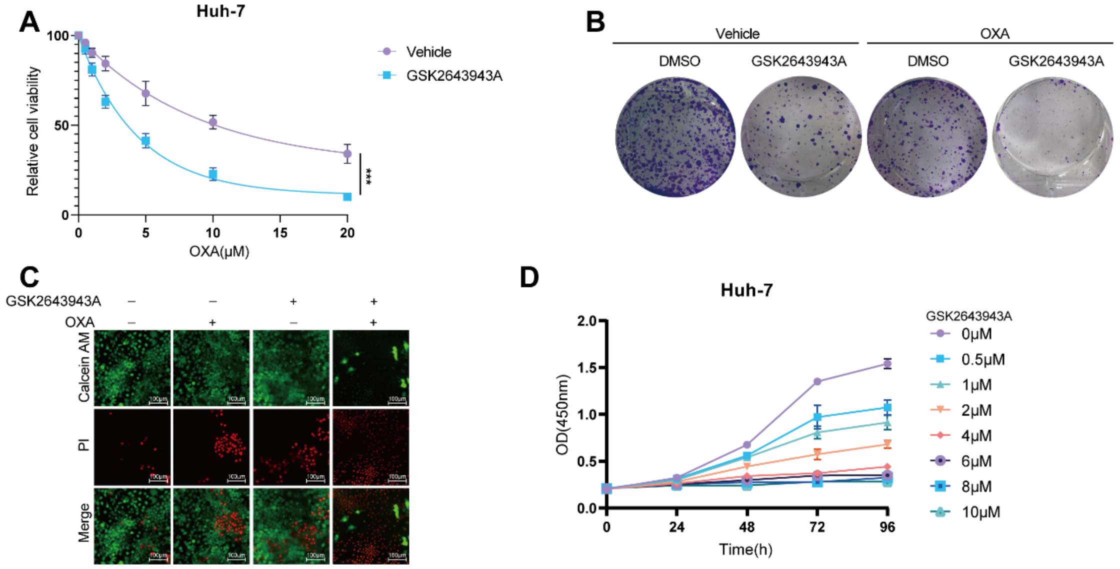

Figure S4. Inhibition USP20 by GSK2643943A suppresses OXA resistance of HCC cells. (A). The survival percentage of HCC cells treated with increasing concentration of OXA for 48h. (Each group contained 3 replicates). (B). Crystal violet staining of HCC cells treated with OXA. (C). Calcein/PI staining of HCC cells treated with OXA (10 μM) for 48h. (D). GSK2643943A treatment inhibited HCC proliferation. (Each group contained 3 replicates).

Results shown are representative of 3 independent experiments. Data are represented as mean ± SD of biological triplicates.*, *P value < 0.05; **, P value < 0.01; ***, P value < 0.001.*

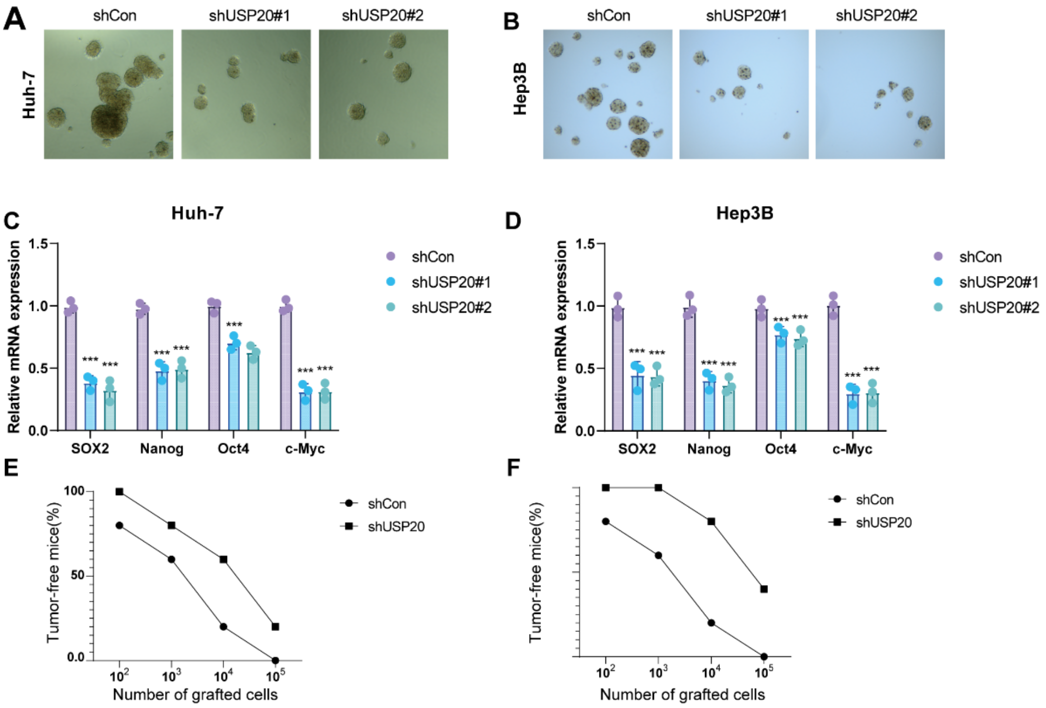

Cell population	Frequency of tumorigenic cells (95% CI)	P value
shCon	1/3782 (1/10127-1/1412)	
ShUSP20	1/37914 (1/101319-1/14187)	<0.001

Cell population	Frequency of tumorigenic cells (95% CI)	P value
shCon	1/3782 (1/10127-1/1412)	
ShUSP20	1/37914 (1/101319-1/14187)	<0.001

Figure S5. USP20 enhances tumorigenic capacity of HCC stem cells. (A, B). Sphere formation assay of USP20-knockdown cells and control cells. (C, D). Depletion of USP20 decreased the expression of pluripotent factors in HCC cells as assessed by quantitative real-time PCR. (E, F). Tumorigenic cell frequency in USP20-knockdown cells and control cells was determined with limiting dilution assays. Results shown are representative of 3 independent experiments. Data are represented as mean ± SD of biological triplicates. *, *P value < 0.05; **, P value < 0.01; ***, P value < 0.001.*

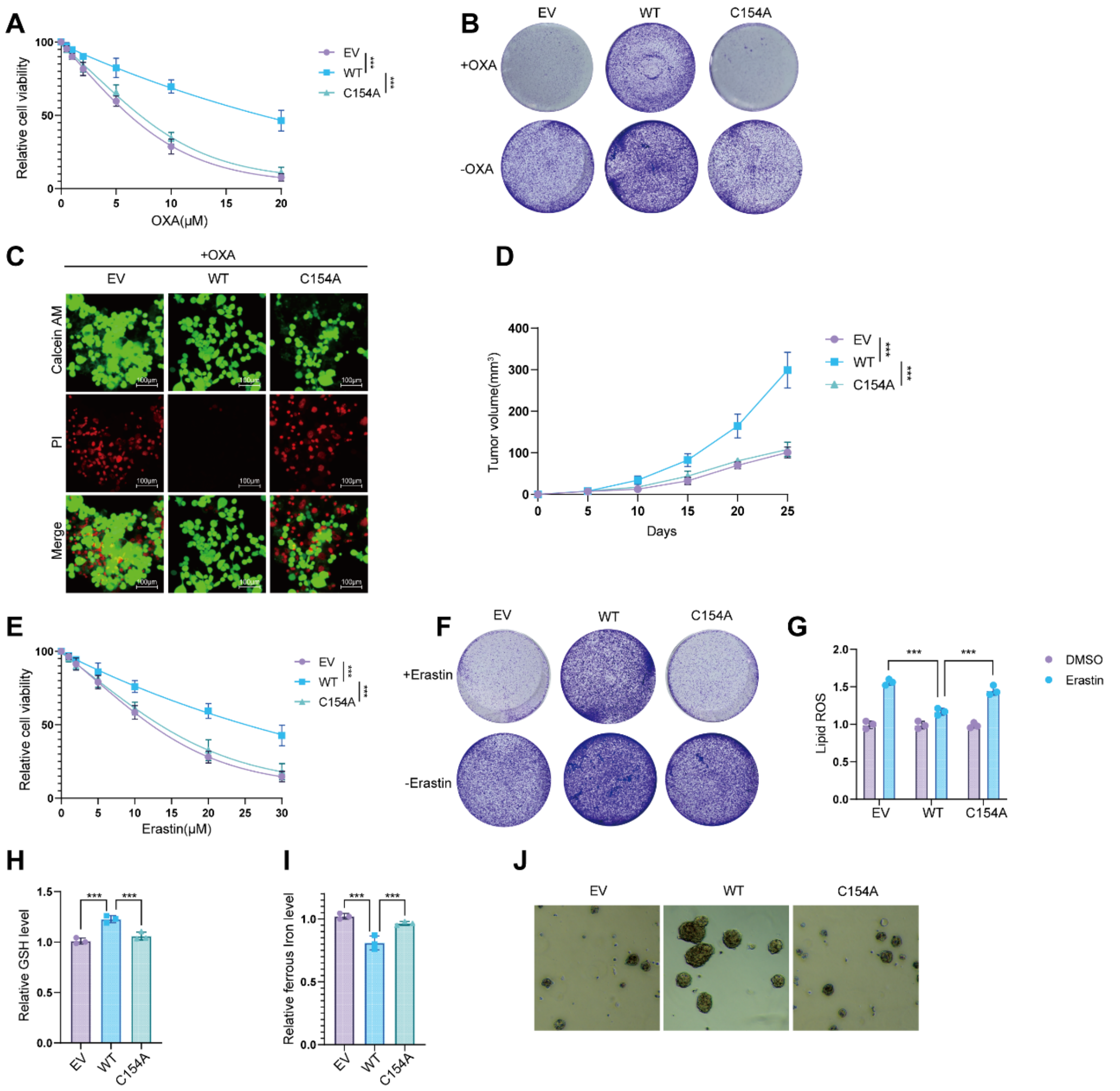

Figure S6. The deubiquitylation activity site of USP20 is necessary to regulate the OXA resistance, ferroptosis and stem-like properties in primary HCC cells. (A). The survival percentage of primary HCC cells treated with increasing concentration of OXA. (Each group contained 3 replicates). (B). Crystal violet staining of primary HCC cells treated with OXA. (C). Calcein/PI staining of primary HCC cells treated with OXA. (D). In vivo xenografts generated from primary HCC cells expressing an empty vector, USP20-WT, or USP20^{C154A} and treated with OXA. 1×10^6 primary HCC cells were injected to the right dorsal flank of each mouse (n=6). After the tumors reached approximately 50 mm^3, the mice were treated with OXA (5 mg/kg, twice a week). Tumor sizes were measured every 5 days until the end of the experiment. (E). The relative cell viability of primary HCC cells treated with the indicated dosage of erastin. (Each group contained 3 replicates). (F). Crystal violet staining of primary HCC cells

treated with erastin. (G-I). Lipid ROS (G), GSH levels (H) and ferrous iron levels (I) were measured in primary HCC cells. (J). Sphere formation assay of primary HCC cells. Results shown are representative of 3 independent experiments. Data are represented as mean ± SD of biological triplicates.*, *P value < 0.05; **, P value < 0.01; ***, P value < 0.001.*

A

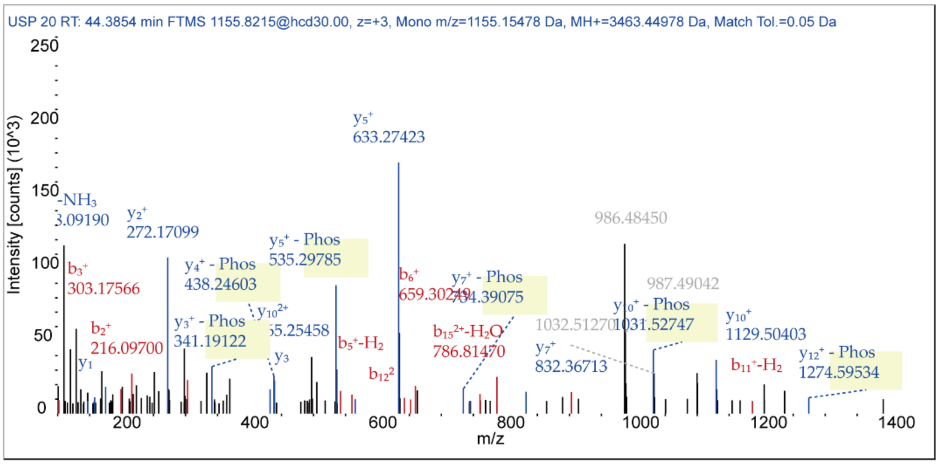

B

Identification of USP20-interacting proteins via mass spectrometry			
Accession	Protein name	Number of unique peptides matched	Coverage [%]
Q9Y2K6	USP20	32	45
Q13535	ATR	3	3

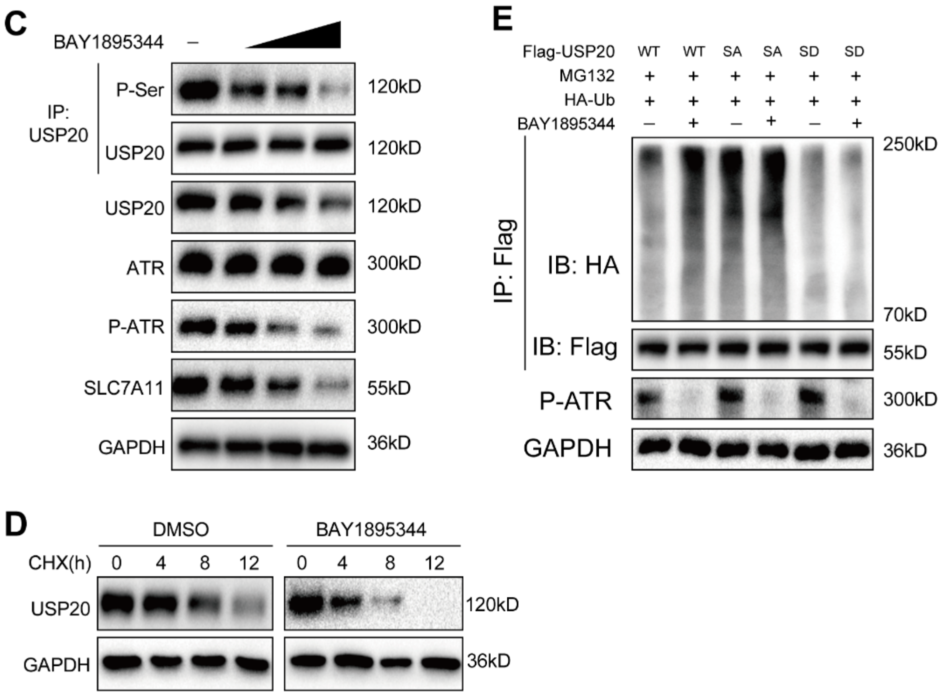

Figure S7. ATR phosphorylates USP20. (A). Mass spectrometry analysis revealed lysine residue S132 and S368 of USP20 were phosphorylated in Huh-7 cells. (B). Mass spectrometry assay of USP20-associated proteins in Huh-7 cells was performed, and the specific interactive information between USP20 and ATR was shown. (C). Co-immunoprecipitation analysis of USP20 serine phosphorylation in Huh-7 cells that

were treated with BAY1895344. (D). Inhibition of ATR by BAY1895344 affected the turnover of USP20. Huh-7 cells were treated BAY1895344. Cells were then treated with CHX for the indicated time, and the expression of USP20 was analyzed by western blotting. (E). HEK293T cells transfected with HA-ubiquitin and Flag-USP20 or indicated Flag-USP20 mutant plasmids were treated with BAY1895344. After treatment with 10 μM MG132 for 6 h, cell lysates were subjected to ubiquitination assay and the ubiquitination level of USP20 was detected by HA antibody.

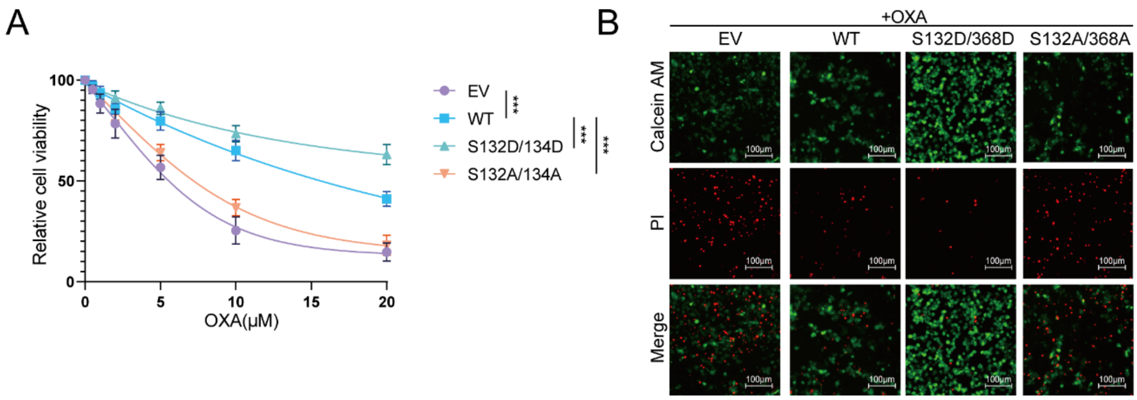

Figure S8. USP20 phosphorylation promotes OXA resistance. (A). HCC primary cells transfected with USP20-WT, S132D/S368D or S132A/S368A were treated with increasing concentration of OXA. The survival percentage of HCC cells were measured using CCK8. (Each group contained 3 replicates). (B). Calcein/PI staining of HCC primary cells treated with OXA.

Results shown are representative of 3 independent experiments. Data are represented as mean ± SD of biological triplicates.*, *P value < 0.05; **, P value < 0.01; ***, P value < 0.001.*

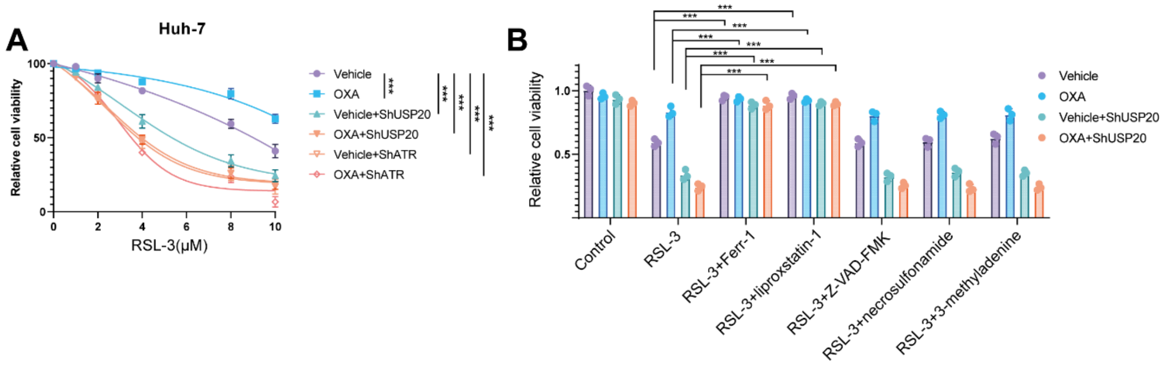

Figure S9. OXA induced DNA damage increases ferroptosis resistance of HCC cells dependent on USP20 (A). The relative cell viability of primary HCC cells were treated with the indicated dosage of RSL-3. (Each group contained 3 replicates). (B). CCK8 assay showing the response of HCC cells were treated with erastin (20 μM) or RSL-3 (5 μM) in the absence or presence of ferrostatin-1 (1 μM), liproxstatin-1 (1 μM), Z-VAD-FMK (10 μM), necrosulfonamide (0.5 μM), or 3-methyladenine (250 μM).

Results shown are representative of 3 independent experiments. Data are represented as mean ± SD of biological triplicates.*, *P value < 0.05; **, P value < 0.01; ***, P value < 0.001.*
